# Cryoballoon Ablation in Older Patients With Heart Failure: Insights From a Large-Scale Multicenter Clinical Study

**DOI:** 10.1016/j.jacadv.2025.102244

**Published:** 2025-10-29

**Authors:** Hiroyuki Miyazawa, Satoshi Yanagisawa, Hirohiko Suzuki, Yukihiko Yoshida, Itsuro Morishima, Yasunori Kanzaki, Shinji Ishikawa, Yosuke Kamikubo, Hiroyuki Kato, Yoshiaki Mizutani, Satoshi Okumura, Yosuke Murase, Kosuke Nakasuka, Shunichiro Warita, Satoru Sekimoto, Yoshio Takemoto, Nobuhiro Takasugi, Shiou Ohguchi, Michiharu Senga, Kenichiro Yokoi, Monami Ando, Ryo Watanabe, Yasuhiro Ogura, Noriyuki Suzuki, Junya Funabiki, Rei Shibata, Yasuya Inden, Toyoaki Murohara

**Affiliations:** a Department of Cardiology, Nagoya University Graduate School of Medicine, Nagoya, Japan; b Department of Cardiology, Ogaki Municipal Hospital, Ogaki, Japan; c Department of Cardiology, Japanese Red Cross Aichi Medical Center Nagoya Daini Hospital, Nagoya, Japan; d Department of Advanced Cardiovascular Therapeutics, Nagoya University Graduate School of Medicine, Nagoya, Japan; e Department of Cardiology, Cardiovascular Center, Anjo Kosei Hospital, Anjo, Japan; f Department of Cardiology, Toyota Memorial Hospital, Toyota, Japan; g Division of Cardiology, Japan Community Healthcare Organization Chukyo Hospital, Nagoya, Japan; h Department of Cardiology, Yokkaichi Municipal Hospital, Yokkaichi, Japan; i Department of Cardiology, Konan Kosei Hospital, Konan, Japan; j Department of Cardiology, Komaki City Hospital, Komaki, Japan; k Department of Cardiology, Nagoya City University Graduate School of Medical Sciences, Nagoya, Japan; l Department of Cardiology, Gifu Prefectural General Medical Center, Gifu, Japan; m Department of Cardiology, Nagoya City University East Medical Center, Nagoya, Japan; n Department of Cardiology, Gifu Prefectural Tajimi Hospital, Tajimi, Japan; o Division of Cardiovascular Medicine, Gifu University Hospital, Gifu, Japan; p Department of Cardiology, Kasugai Municipal Hospital, Kasugai, Japan; q Department of Cardiology, Kuwana City Medical Center, Kuwana, Japan; r Department of Cardiology, Kainan Hospital, Yatomi, Japan

**Keywords:** atrial fibrillation, catheter ablation, cryoballoon ablation, heart failure, older adults

## Abstract

**Background:**

Cryoballoon ablation (CBA) achieves satisfactory outcomes in patients with atrial fibrillation (AF). However, its feasibility in older patients with heart failure (HF) remains unclear.

**Objectives:**

This study examined the efficacy and prognosis of CBA in older patients with HF.

**Methods:**

Among 3,655 patients undergoing CBA at 17 institutions, 549 patients (185 with ≥75 years [older], and 364 with <75 years [younger]) diagnosed with HF were analyzed. Recurrence, mortality, and HF hospitalization after CBA between the older and younger groups were compared using Kaplan-Meier curves and Simon-Makuch analyses. Changes in left ventricular ejection fraction (LVEF) on echocardiography and B-type natriuretic peptide levels were evaluated using linear regression analysis. Major procedural complications included stroke, cardiac tamponade, phrenic nerve injury, prolonged hospitalization, and others.

**Results:**

Most (78%) patients had a preserved LVEF of ≥50%. Major procedural complications were similar in the older and younger groups (3.2% vs 4.7%; *P* = 0.670). The recurrence-free survival and mortality rates were comparable between the 2 groups during a median follow-up period of 21.5 (12.0-37.0) months. The HF hospitalization rate was higher in the older group (4.0 vs 1.5 per 100 patient-years; *P* = 0.008). In the older group, LVEF and B-type natriuretic peptide levels improved after ablation (from 57.4% to 60.0%, and 173 [113-292] to 87.8 [42-218] pg/mL). AF recurrence and HF hospitalization were closely linked, with most hospitalizations occurring after a year postablation.

**Conclusions:**

CBA for AF in older patients with HF is feasible and improves cardiac parameters; however, specific care is required owing to this population’s unique characteristics.

Atrial fibrillation (AF) is a well-recognized condition with increased prevalence in the older population. With the projected increase of 22% in the older population by 2050,^1^ the number of patients with AF is expected to increase accordingly. AF management in older patients is challenging owing to decreased medication adherence, comorbidities, frailty, and life expectancy. Specifically, heart failure (HF) is frequently complicated with AF and influences their respective progression.^2^ Since an increasing number of older patients with both AF and HF has significant economic burden in an advanced society, effective and feasible treatments that improve their prognosis are urgently required.

Treatment approaches for AF in older patients with HF are limited because most antiarrhythmic drugs are contraindicated or poorly tolerated. Rhythm control therapy for AF in such populations has shifted toward catheter ablation therapy, possibly because of the increased opportunities for older patients with HF. Recent large-scale multicenter studies have demonstrated the efficacy of cryoablation for AF in patients with HF, implying the potential of cryoballoon as an approach to treat AF and HF, with a high success rate and few complications.^3^^,^^4^ However, it is unclear whether, similar to younger patients, older patients with HF would benefit from catheter ablation, considering the exclusion or underrepresentation of the older population in past clinical trials.^5^ Currently, no systematic study has focused on the outcomes and prognosis of older patients with HF after AF catheter ablation. It is essential to explore this population more thoroughly, especially given the increasing number of older patients undergoing AF ablation and recent worldwide trend of HF incidence.

Thus, this study was conducted to evaluate the efficacy and safety of cryoballoon ablation (CBA) in older patients with AF and HF in a post hoc analysis of a large-scale multicenter study. We further compared the outcomes and prognosis after ablation in these patients with those in younger patients with HF, elucidating the underlying factors and characteristics associated with adverse events that are specific to older adults.

## Methods

### Study population

The study population was retrospectively recruited from a database of patients with HF across 17 institutions in Japan who underwent CBA for AF. The protocol for recruiting the study participants has been described previously.^3^^,^^6^ All patients with HF who underwent CBA for AF during their first-time ablation from 2014 to 2019 at the participating hospitals were included in the analysis. The population was further divided into older (≥75 years of age) and younger (<75 years) groups at the ablation index date.^1^^,^^7^^,^^8^ The indications for catheter ablation for AF complied with recent guidelines.^9^ CBA was selected at the operator’s discretion at each institution according to patient characteristics and anatomy of the left atrium (LA) and pulmonary vein. Informed consent for the study procedure was obtained from all the patients. The study protocol was approved by each institution’s ethics committee, and the study was performed in accordance with the principles of the Declaration of Helsinki.

### Definition of HF

HF was defined as a previous history of hospitalization or diagnosis of HF, a reduced left ventricular ejection fraction (LVEF) of ≤40% at baseline, continuous administration of diuretics to control congestive HF, and elevated B-type natriuretic peptide (BNP) levels, with symptoms and signs of breathlessness, fatigue, and leg edema. The study did not include only patients with high BNP levels; rather, each patient had symptoms associated with the presence of HF. The chief investigator decided the final inclusion of patients with HF by reviewing the patients’ medical records at each institution. The etiology of HF was determined to be the most likely cause of HF based on examinations and assessments performed by the investigator on each patient.

### Catheter ablation procedure

Before the cryoballoon procedure, transesophageal echocardiography and/or contrast-enhanced computed tomography was performed to exclude the presence of LA thrombus and clarify the anatomy of the LA and pulmonary vein. Oral anticoagulants were administered for >3 weeks before ablation. Periprocedural management of anticoagulation and antiarrhythmic drug prescriptions was determined by each institution.^3^^,^^10^ The ablation procedure was performed with the patient under minimal-to-moderate sedation. The activated clotting time was maintained between 300 and 350 s via heparin infusion adjustment during the procedure. After transseptal puncture, a 28-mm second-generation cryoballoon catheter (Arctic Front Advance, Medtronic Inc) was positioned at the pulmonary vein ostium using a 12-F steerable sheath (Flex Cath Advance, Medtronic Inc). A duty cycle cryoapplication of 180 to 240 s was repeatedly performed at each pulmonary vein, while monitoring the pulmonary vein potentials with a spiral-mapping catheter (Achieve, Medtronic Inc). If the pulmonary vein potential remained after repeated CBA, touch-up ablation was performed using a radiofrequency or cryoablation catheter. Additional linear and substrate ablations were performed at the discretion of the attending operators where necessary. Major procedural complications were defined as stroke, transient ischemic attack, cardiac tamponade, phrenic nerve injury lasting more than 1 month, pseudoaneurysm, arteriovenous fistula, pneumothorax, and prolonged hospitalization due to worsening HF.

### Follow-up and outcome assessment

After discharge, the patients were followed up at the outpatient clinic of each institution. At each follow-up visit, patients underwent surface 12-lead electrocardiography or Holter monitoring examinations and were asked about any symptoms related to the presence of arrhythmia. Where necessary, additional electrocardiography and Holter monitoring examinations were scheduled with a short follow-up duration when patients reported palpitations with suspected recurrence. The exact monitoring of recurrence was not predefined; rather, it was conducted according to the respective institutions’ standard of clinical practice, including device interrogation data and insertable cardiac monitoring.^6^ AF recurrence was defined as atrial tachyarrhythmias lasting >30 s during the examination after the 3-month blanking period, regardless of antiarrhythmic drug administration. This study also assessed the clinical outcomes of all-cause death and HF hospitalization (first admission) in each patient following ablation. Hospitalization solely due to AF was not considered an endpoint. The composite endpoint was defined as the composite of all-cause death and HF hospitalizations. The latest follow-up echocardiographic data on cardiac function and changes in BNP levels after ablation were evaluated. We compared the outcomes and prognosis between the older and younger groups and further evaluated the outcomes based on age, recurrence, etiologies of HF, NYHA functional class, and baseline LVEF. All patient characteristics, procedural data, and outcomes were retrieved from the patients’ medical records and procedural databases of each institution.

### Statistical analysis

Continuous data are presented as mean ± SD or median (IQR). Comparisons of the differences in the baseline characteristics between patients aged ≥75 and <75 years were analyzed using Student’s *t*-test for parametric data and the Mann-Whitney *U* test for nonparametric data. Categorical variables were compared using the chi-squared or Fisher exact test. Differences in the baseline characteristics among more than 2 different older groups, categorized by baseline LVEF or NYHA functional class, were analyzed using one-way analysis of variance, the Kruskal-Wallis test, and the chi-squared test, as appropriate. Differences between the baseline and follow-up BNP levels and LVEF were compared using linear regression analysis, taking into account the duration between measurements. Event-free survival rates were estimated using Kaplan-Meier curve analysis. Simon-Makuch analysis was also used to adjust for a time-dependent event, the recurrence factor. The associated value of each factor was first evaluated using univariable Cox regression analysis. Factors with a *P* value of <0.05 in the univariable analysis were entered into the multivariable Cox regression model using the backward stepwise method to identify independent associated factors. Multivariable Cox regression models with a time-dependent covariate of recurrence and a mixed-effects Cox regression model with institution variation were also analyzed. The optimal model of variables was selected by Akaike's Information Criterion minimization method.^11^ An adjusted Cox proportional hazards model was used to estimate the HRs for outcomes with associated 95% CIs, along with the continuous value of age in years. The Fine–Gray model was used to estimate HRs of the outcomes, accounting for competing risks of death. Statistical significance was set at *P* < 0.05. All analyses were performed using the SPSS software (version 29.0, IBM Corporation) and R (version 4.3.2).

## Results

### Baseline patient characteristics and procedural results

Among the 3,655 patients who underwent CBA for AF at 17 institutions during the study period, 549 (15%) were diagnosed with HF preoperatively (185 patients, aged ≥75 years; 364 patients aged <75 years). The baseline characteristics and examination results of the total population and patients aged ≥75 and < 75 years are shown in Table 1. In the older group, the mean age was 79.1 ± 3.7 years, and 42% of the patients were men. Majority of them had paroxysmal AF (72%), although 4 (2.2%) patients with long-standing persistent AF were also included. While the mean LVEF of the older population was preserved at 58.1% ± 11.5%, 15 (8.1%) patients had reduced cardiac function, defined as an LVEF of ≤40%. In most (70%) patients, the etiology of HF was suspected to be tachycardia-induced cardiomyopathy (TIC). The older group had a higher proportion of patients with NYHA functional class II and hypertension, as well as a greater prevalence of diuretic use, than the younger group. The older group also exhibited a smaller left ventricular diameter, a higher LVEF, and elevated BNP levels than those exhibited by the younger group. A significantly lower prevalence of an LVEF of ≤40% and a higher prevalence of an LVEF of ≥50% was observed in the older group than in the younger group.

**Table 1:** Comparison of Baseline Characteristics Between the Older and Younger Groups

	Patients With HF and AF (n = 549)	Patients With HF Aged ≥75 Years (n = 185)	Patients With HF Aged <75 Years (n = 364)	*P* Value^a^
Age, y	69.3 ± 10.0	79.1 ± 3.7	64.3 ± 8.4	<0.001
Male	315 (57%)	78 (42%)	237 (65%)	<0.001
Body weight, kg	62.1 ± 12.5	56.1 ± 10.1	65.2 ± 12.4	<0.001
Body mass index, kg/m^2^	23.8 ± 3.9	22.9 ± 3.4	24.2 ± 4.0	<0.001
Duration of AF, y	0.5 (0.3-2.0)	0.5 (0.3-2.0)	0.5 (0.3-2.0)	0.670
AF type				
Paroxysmal	391 (71%)	134 (72%)	257 (71%)	0.655
Persistent	134 (24%)	74 (25%)	87 (24%)	0.655
Long-standing persistent	24 (4.4%)	4 (2.2%)	20 (5.5%)	0.071
Antiarrhythmic drugs				
Class I	110 (20%)	44 (24%)	66 (18%)	0.118
Class III	90 (16%)	26 (14%)	64 (18%)	0.291
NYHA functional class	1.4 ± 0.6	1.4 ± 0.5	1.3 ± 0.6	0.294
I	381 (69%)	118 (64%)	263 (72%)	0.042
II	149 (27%)	63 (34%)	86 (24%)	0.009
III and IV	19 (3.5%)	4 (2.2%)	15 (4.1%)	0.235
Comorbidity				
Hypertension	343 (63%)	133 (72%)	210 (58%)	0.001
Diabetes mellitus	119 (22%)	39 (21%)	80 (22%)	0.809
Coronary artery disease	61 (11%)	18 (9.7%)	43 (12%)	0.463
Stroke/TIA	61 (11%)	22 (12%)	39 (11%)	0.678
Hemodialysis	18 (3.3%)	3 (1.6%)	15 (4.1%)	0.120
Echocardiographic data				
Left atrial diameter, mm	40.5 ± 6.1	40.2 ± 6.1	40.7 ± 6.1	0.324
LVEDD, mm	48.4 ± 8.7	46.1 ± 6.5	49.6 ± 9.5	<0.001
LVESD, mm	33.7 ± 8.2	31.7 ± 7.0	34.8 ± 8.5	<0.001
LVEF, %	56.2 ± 12.8	58.1 ± 11.5	55.2 ± 13.3	0.008
LVEF ≤40%	73 (13%)	15 (8.1%)	58 (16%)	0.011
LVEF 40%-49%	82 (15%)	25 (14%)	57 (16%)	0.505
LVEF ≥50%	394 (72%)	145 (78%)	249 (68%)	0.014
CHADS_2_ score	2.3 ± 1.1	3.1 ± 1.0	1.9 ± 1.0	<0.001
CHA_2_DS_2_-VASc score	3.5 ± 1.5	4.7 ± 1.1	2.9 ± 1.3	<0.001
Laboratory data				
Creatinine clearance, mL/min	63.4 ± 28.5	47.5 ± 14.4	71.5 ± 30.4	<0.001
BNP levels, pg/dL	154 (104-259)	174 (120-299)	149 (99.9-231)	0.002
DOAC	483 (88%)	163 (88%)	320 (88%)	0.947
History of device implantation				
Pacemaker	24 (4.4%)	13 (7.0%)	11 (3.0%)	0.030
ICD	12 (2.2%)	2 (1.1%)	10 (2.7%)	0.354
CRT	4 (0.7%)	2 (1.1%)	2 (0.5%)	0.606
Medications				
ACEI/ARB	266 (49%)	93 (50%)	173 (48%)	0.543
ARNI	0 (0%)	0 (0%)	0 (0%)	NA
Beta-blocker	372 (68%)	115 (62%)	257 (71%)	0.045
Spironolactone	105 (19%)	39 (21%)	66 (18%)	0.406
SGLT-2 inhibitor	15 (2.7%)	6 (2.2%)	9 (2.5%)	0.612
Diuretic	208 (38%)	82 (44%)	126 (35%)	0.027
Etiology of HF				
Tachycardia-induced cardiomyopathy	365 (67%)	129 (70%)	236 (65%)	0.251
Ischemic cardiomyopathy	100 (18%)	27 (15%)	73 (20%)	0.117
Dilated cardiomyopathy	15 (2.7%)	3 (1.6%)	12 (3.3%)	0.255
Hypertrophic cardiomyopathy	18 (3.3%)	5 (2.7%)	13 (3.6%)	0.589
Valvular heart disease	25 (4.6%)	13 (7.0%)	12 (3.3%)	0.048
Sarcoidosis	0 (0%)	0 (0%)	0 (0%)	N/A
Amyloidosis	2 (0.4%)	1 (0.5%)	1 (0.3%)	0.561
Other	24 (4.4%)	7 (3.8%)	17 (4.7%)	0.631

ACEI = angiotensin-converting enzyme inhibitor; AF = atrial fibrillation; ARB = angiotensin receptor blocker; ARNI = angiotensin receptor neprilysin inhibitor; BNP = B-type natriuretic peptide; CRT = cardiac resynchronization therapy; DOAC = direct oral anticoagulant; HF = heart failure; ICD = implantable cardioverter-defibrillator; LVEDD = left ventricular end-diastolic diameter; LVESD = left ventricular end-systolic diameter; LVEF = left ventricular ejection fraction; SGLT = sodium glucose cotransporter; TIA = transient ischemic attack.

Values are mean ± SD or n (%).

a ≥75 years vs <75 years.

A comparison of the procedural details between the 2 groups is shown in Table 2. Pulmonary vein isolation was performed in all patients. Few (6.2%) patients in both groups underwent additional LA ablation. No significant differences in major complications were observed between the older and younger groups (3.2% vs 4.7%; *P* = 0.430).

**Table 2:** Procedural Details of Patients With HF Aged ≥75 or <75 Years

	Patients With HF and AF (n = 549)	Patients With HF Aged ≥75 Years (n = 185)	Patients With HF Aged <75 Years (n = 364)	*P* Value^a^
Successful PVI	549 (100%)	185 (100%)	364 (100%)	N/A
Touch-up for incomplete PVI of cryoballoon	156 (28%)	50 (27%)	106 (29%)	0.607
Additional ablations				
Linear ablations in LA (roof, bottom, and mitral isthmus lines)	34 (6.2%)	12 (6.5%)	22 (6.0%)	0.839
Superior vena cava isolation	5 (0.9%)	1 (0.5%)	4 (1.1%)	0.453
Cavotricuspid isthmus ablation	410 (75%)	142 (77%)	268 (74%)	0.425
CFAE ablation	0 (0%)	0 (0%)	0 (0%)	N/A
Other ablations for another arrhythmias	7 (1.3%)	2 (1.1%)	5 (1.4%)	0.562
Session time, min (from puncture to session end)	134.1 ± 40.5	133.2 ± 40.9	134.6 ± 40.3	0.730
Major complications				
Overall	23 (4.2%)	6 (3.2%)	17 (4.7%)	0.430
Stroke	1 (0.1%)	1 (0.5%)	0 (0%)	0.337
Transient ischemic attack	0 (0%)	0 (0%)	0 (0%)	N/A
Cardiac tamponade	4 (0.7%)	2 (1.1%)	2 (0.5%)	0.414
Phrenic nervous injury (persisting >1 month)	10 (1.8%)	2 (1.1%)	8 (2.2%)	0.507
Pseudoaneurysm	3 (0.5%)	1 (0.5%)	2 (0.5%)	0.736
Arteriovenous fistula	3 (0.5%)	0 (0%)	3 (0.8%)	0.291
Pneumothorax	0 (0%)	0 (0%)	0 (0%)	N/A
Prolonged hospitalization due to worsened HF	2 (0.4%)	0 (0%)	2 (0.5%)	0.439
Others	2 (0.4%)	2 (1.1%)	0 (0%)	0.439

CFAE = complex fractionated atrial electrogram; LA = left atrium; PVI = pulmonary vein isolation; other abbreviations as in Table 1.

Values are n (%) or mean ± SD.

a ≥75 years vs <75 years.

### Prognosis between the older and younger groups

**Figure 1:**
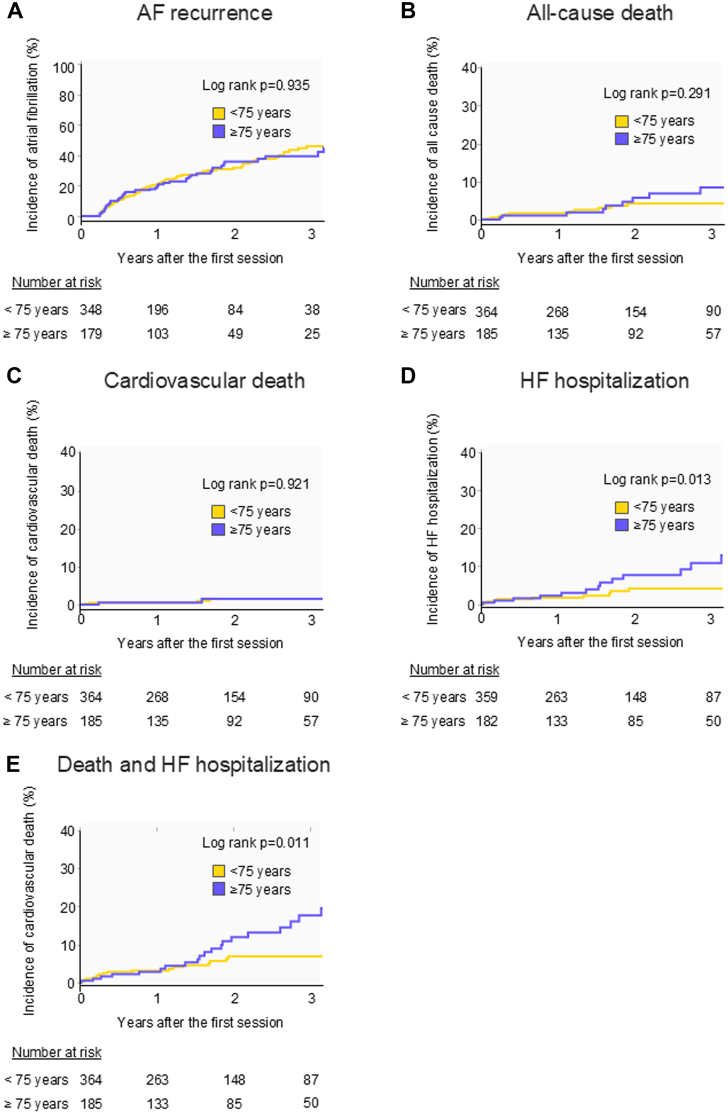
Kaplan-Meier Cumulative Incidence Curves of Prognoses in Patients with HF Aged ≥75 or <75 Years AF recurrence (A), all-cause death (B), cardiovascular death (C), HF hospitalization (D), and death and HF hospitalization (E). AF = atrial fibrillation; HF = heart failure.

During a median follow-up period of 21.5 (12.0-37.0) months, 152 (28%) patients experienced postablation recurrence. Antiarrhythmic drugs were administered to 156 patients during the follow-up. Repeat ablations for recurrence were performed in 19% of the patients (Table 3). Among the older and younger groups, the Kaplan-Meier cumulative incidence curves showed no statistically significant differences in the risk of recurrence, all-cause death, or cardiovascular death (Figure 1A–C, Table 3). However, the older group demonstrated significantly higher HF hospitalization and composite events of death and HF hospitalization rates than that demonstrated by the younger group (Figure 1D, E). The median duration of the time to death after the ablation in 22 patients was 501 (99-702) days. There were no significant differences in the recurrence rates of AF between patients who died and those who did not, in either the older (46% vs 33%; *P* = 0.612) or younger (55% vs 67%; *P* = 0.591) group. Survival curve analysis for the abovementioned outcomes using Simon-Makuch analysis after the adjustment of a time-dependent event, recurrence factor is shown in Supplemental Figure 1.

**Table 3:** Clinical Endpoints During the Follow-Up Period

	Patients With HF and AF (n = 549)	Patients With HF Aged ≥75 Years (n = 185)	Patients With HF Aged <75 Years (n = 364)	*P* Value^a^
Early recurrence^b^	136 (25%)	46 (25%)	90 (25%)	0.930
Recurrence^b^	152 (28%)	53 (30%)	99 (28%)	0.751
Repeat session for the recurrence^b^	102 (19%)	32 (17%)	70 (19%)	0.572
AAD therapy after ablation^b^	156 (28%)	52 (29%)	104 (29%)	0.923
Class I	58 (11%)	18 (9.7%)	40 (11%)	0.657
Class III	98 (18%)	34 (18%)	64 (18%)	0.807
All-cause death	22 (4.0%)	10 (5.4%)	12 (3.3%)	0.234
(per 100 patient-years)	1.92 (1.12-2.73)	2.50 (0.95-4.05)	1.61 (0.70-2.52)	
Cardiovascular death	6 (1.1%)	2 (1.1%)	4 (1.1%)	0.674
(per 100 patient-years)	0.52 (0.10-0.94)	0.50 (0.00-1.19)	0.53 (0.01-1.06)	
HF hospitalization^b^	26 (4.8%)	15 (8.2%)	11 (3.1%)	0.008
(per 100 patient-years)	2.35 (1.44-3.25)	3.96 (1.96-5.97)	1.51 (0.61-2.40)	
Composite endpoint^b^	41 (7.6%)	22 (12%)	19 (5.3%)	0.005
(per 100 patient-years)	3.70 (2.57-4.84)	5.82 (3.38-8.25)	2.61 (1.43-3.78)	

AAD = antiarrhythmic drugs; other abbreviations as in Table 1.

Values are n (%) or mean ± SD.

a ≥75 years vs <75 years.

b Early recurrence, recurrence, repeat sessions for recurrence, AAD therapy after ablation, HF hospitalization, and major adverse event data are available for 543, 527, 548, 541, 541, and 541 patients, respectively. The composite endpoint was defined as the composite of all-cause death and HF hospitalization.

**Figure 2:**
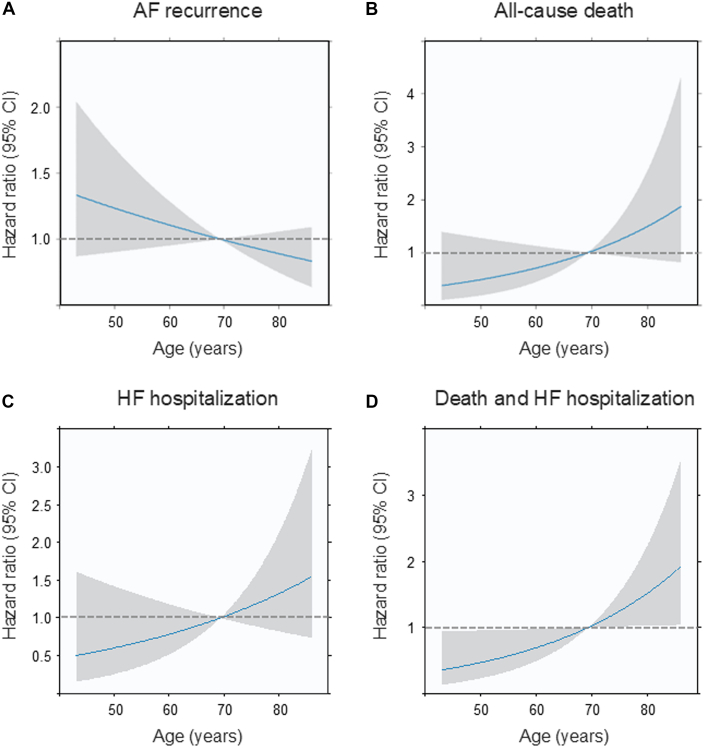
Estimated Risk of Outcomes After Cryoablation According to Age AF recurrence (A), all-cause death (B), HF hospitalization (C), and death and HF hospitalization (D). The estimated risks of adverse events were analyzed with age as a continuous variable. The solid blue line illustrates the estimated HR, with 95% CIs depicted as shaded areas. Abbreviations as in Figure 1.

Although the estimated HR for recurrence decreased continuously with advancing age (Figure 2A), the HR for all-cause death, HF hospitalization, and composite events of death and HF hospitalization increased incrementally (Figure 2B–D). The estimated risks of AF recurrence and HF hospitalization by age, considering the competing risk of death, showed a decreased risk of recurrence and an increased risk of HF hospitalization with advancing age (Supplemental Figure 2).

### Changes in BNP levels and echocardiographic findings following ablation

**Figure 3:**
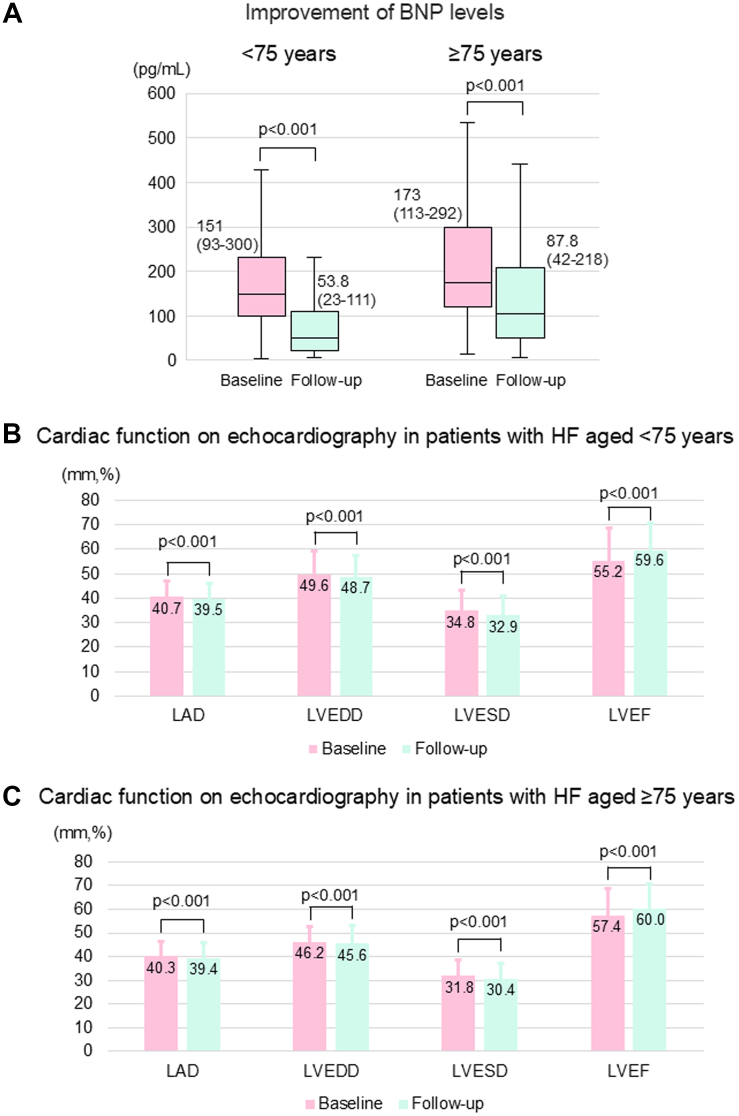
Changes in BNP Levels and Echocardiographic Findings From Baseline to Postablation Regarding the association between BNP levels (A) and echocardiographic data (B and C) before and after treatment, we conducted a linear regression analysis using post-treatment value as the outcome variable, and pretreatment value and the measurement interval as covariates, taking into account the duration between measurements. BNP levels were logarithmically transformed for analysis. BNP = brain natriuretic peptide; LAD = left atrial diameter; LVEDD = left ventricular end-diastolic diameter; LVESD = left ventricular end-systolic diameter; LVEF = left ventricular ejection fraction; other abbreviation as in Figure 1.

BNP levels and echocardiographic findings were obtained during the follow-up period from 381 and 440 patients with HF, respectively. The BNP levels significantly decreased in both groups during a median follow-up of 14.7 (7.4-29.5) months after ablation (Figure 3A). In both the younger and older groups, all 4 parameters (LA diameter, LV end-diastolic diameter, LV end-systolic diameter, and LVEF) showed significant improvement (Figures 3B and 3C).

### Prognosis of the older group

**Figure 4:**
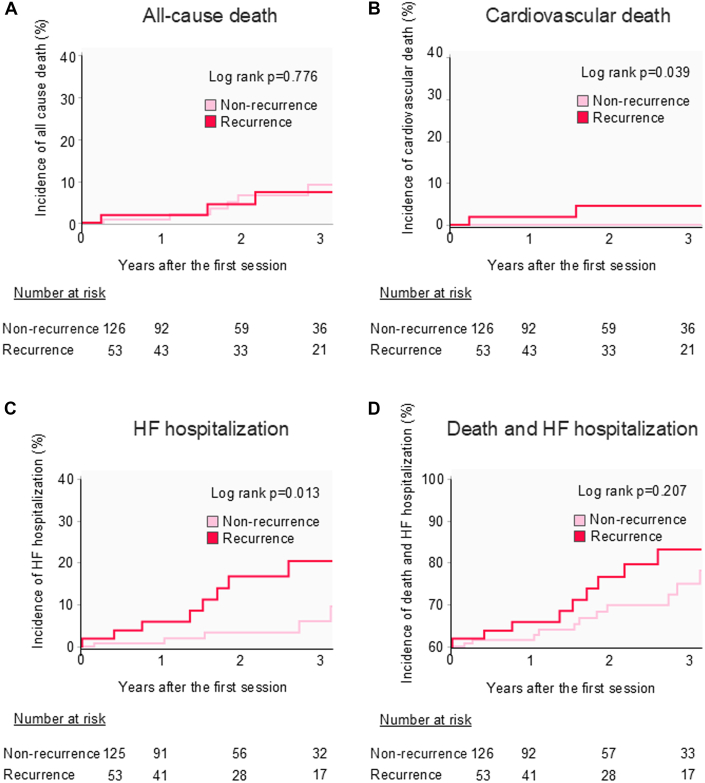
Kaplan-Meier Survival Curves of Prognoses in Older Patients With or Without Recurrence All-cause death (A), cardiovascular death (B), HF hospitalization (C), and composite endpoint (D). Abbreviation as in Figure 1.

**Figure 5:**
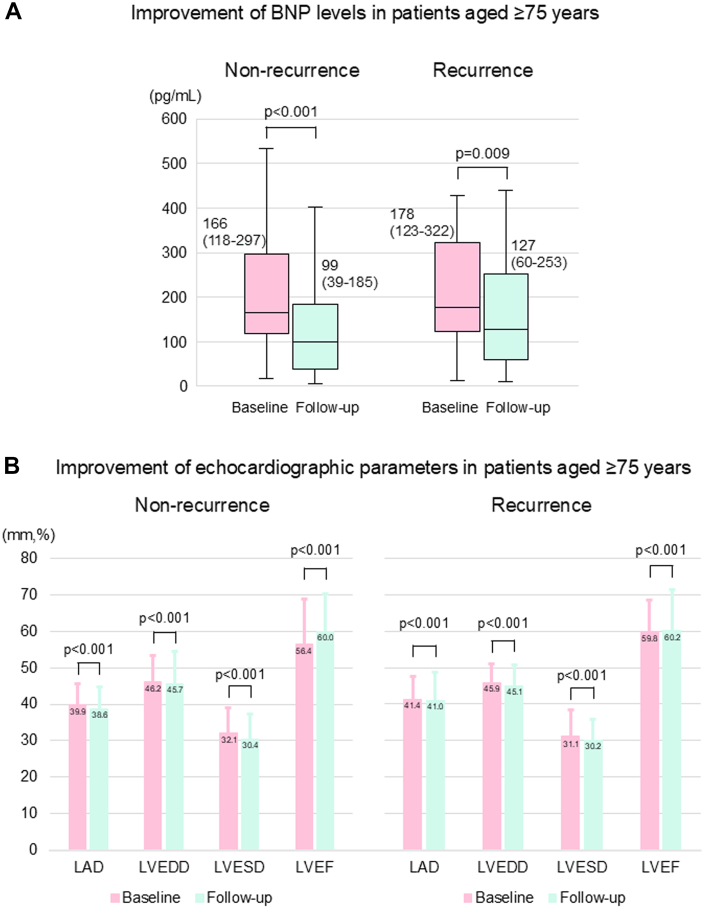
Changes in Parameters From Baseline to Postablation in Older Patients With or Without Recurrence Regarding the association between BNP levels and echocardiographic data before and after treatment, we conducted a linear regression analysis using post-treatment value as the outcome variable, and pretreatment value and the measurement interval as covariates, taking into account the duration between measurements. BNP levels were logarithmically transformed for analysis. BNP levels (A) and echocardiographic findings (B). Abbreviations as in Figure 3.

Among older patients, no significant differences in all-cause death were observed between the recurrence and nonrecurrence subgroups (Figure 4A); however, the recurrence subgroup had a significantly higher occurrence of cardiovascular death and HF hospitalization than that had by the nonrecurrence subgroup after ablation (Figures 4B and 4C). No significant differences were observed between the 2 subgroups in the incidence of the composite endpoint of death and HF hospitalization (Figure 4D). Survival curve analysis for the abovementioned outcomes using Simon-Makuch analysis after adjusting for a time-dependent event; recurrence factor is shown in Supplemental Figure 3. BNP levels decreased significantly both in the nonrecurrence and recurrence subgroups (Figure 5A). Echocardiographic findings revealed significant improvements in all parameters in both subgroups (Figure 5B).

Multivariable Cox proportional hazards analysis showed that baseline BNP level and NYHA functional class were independently associated with the composite endpoints of death and HF hospitalization after ablation in the older group (Table 4). Multivariable Cox hazard models with time-dependent covariates and mixed-effects multivariable Cox hazard model for adjustment of institutional variability are shown in Supplemental Tables 1 and 2. The details of the 4 patients with severe NHYA functional class III/IV in the older group are provided in Supplemental Table 3. All the 4 patients had a baseline LVEF of ≥40%, although their CHA_2_DS_2_-VASc scores were high, reflecting the presence of multiple comorbidities. Another important observation was that recurrence, HF hospitalization, and death occurred long after ablation, often exceeding 1 year postprocedure in these 4 patients.

**Table 4:** Factors Associated With Composite Events of All-Cause Death and HF Hospitalization After Cryoballoon Ablation in Older Patients With HF

	Univariable Analysis		Multivariable Analysis	
	HR (95% CI)	*P* Value	HR (95% CI)	*P* Value
Age, y	1.061 (0.952-1.183)	0.284		
Female sex	0.917 (0.392-2.149)	0.843		
Body mass index, kg/m^2^	1.032 (0.921-1.156)	0.591		
AF duration, y	1.067 (0.963-1.183)	0.215		
Creatinine clearance level, mL/min	0.956 (0.930-0.983)	0.002^a^		
BNP, pg/dL	1.003 (1.002-1.004)	<0.001^a^	1.002 (1.001-1.003)	<0.001
LAD, mm	1.034 (0.965-1.108)	0.337		
LVEF, %	0.974 (0.944-1.005)	0.096		
LVEDD, mm	1.060 (0.996-1.129)	0.066		
CHADS_2_ score	1.532 (1.065-2.203)	0.022^a^	1.418 (0.959-2.098)	0.080
CHA_2_DS_2_-VASc score	1.626 (1.118-2.366)	0.011^a^		
Persistent AF	0.969 (0.379-2.477)	0.948		
NYHA class	2.635 (1.356-5.121)	0.004^a^	1.928 (1.021-3.641)	0.043
Structural heart disease	1.576 (0.680-3.655)	0.289		
Recurrence	1.823 (0.773-4.299)	0.170		
Early recurrence	1.626 (0.681-3.879)	0.274		
History of device implantation	1.899 (0.641-5.627)	0.247		

Data from 182 patients were analyzed.

LAD = left atrial diameter; other abbreviations as in Table 1.

a Variables included in the multivariable model using the backward stepwise method.

### Subgroup analysis based on various viewpoints in the older group

In the older population, Kaplan-Meier survival curves showed no statistically significant differences in recurrence, all-cause, and cardiovascular death, or HF hospitalization between the 2 subtypes of AF (paroxysmal and persistent AF; Supplemental Figure 4). Survival curve analysis for the outcomes mentioned above using Simon-Makuch analysis after adjusting for a time-dependent event, with the recurrence factor, is shown in Supplemental Figure 5.

**Figure 6:**
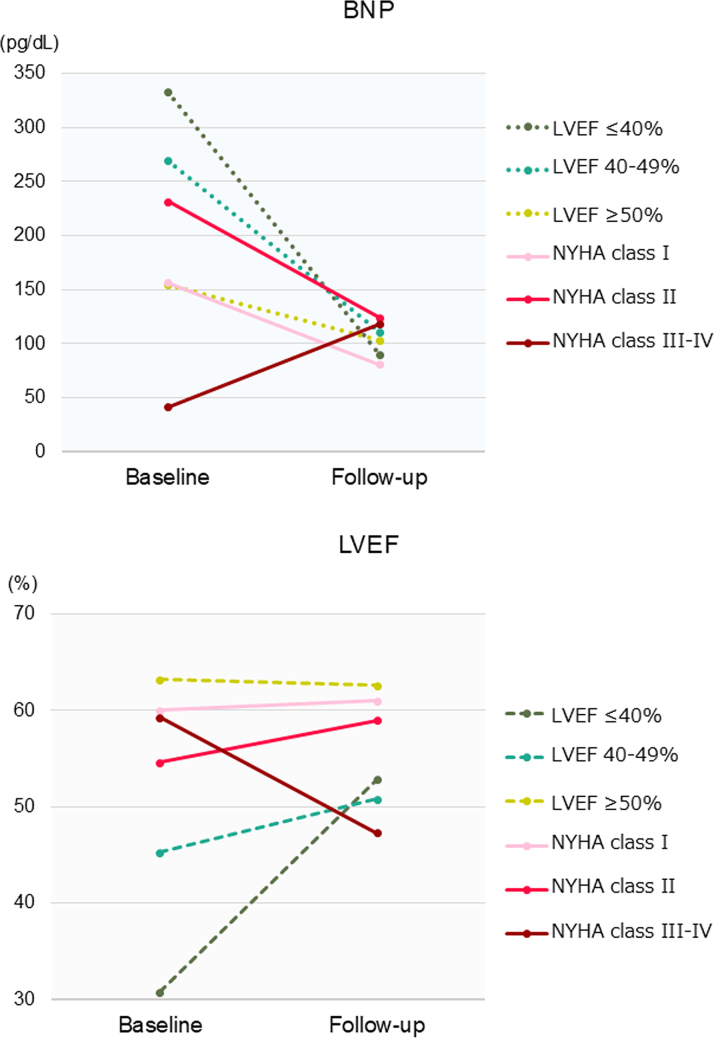
Changes in BNP Levels and LVEF After Ablation by Subgroups of Older Patients Abbreviations as in Figure 3.

The baseline characteristics of the patients with an LVEF of ≤40% (n = 15), LVEF of 40% to 50% (n = 25), and LVEF of ≥50% (n = 145) and those with NYHA functional class I (n = 118), II (n = 63), and III–IV (n = 4) in the older group are presented in Supplemental Tables 4 and 5, respectively. BNP levels and LVEF improved after ablation in most LVEF and NYHA functional class subgroups (Supplemental Table 6, Figure 6). However, patients with NYHA functional classes III and IV exhibited a trend toward worse LVEF and BNP levels after ablation.

### Relationship between recurrence and HF hospitalization in older and younger patients

For all older patients hospitalized for HF after ablation, the duration until hospitalization, presence or absence of AF recurrence, and time to recurrence are shown in Central Illustration. First, the dates of recurrence and HF hospitalization were closely correlated. Multivariable Cox proportional hazards analysis showed that baseline LVEF and AF recurrence were independently associated with HF hospitalization after ablation in the older group (Supplemental Table 7).

**Central Illustration:**
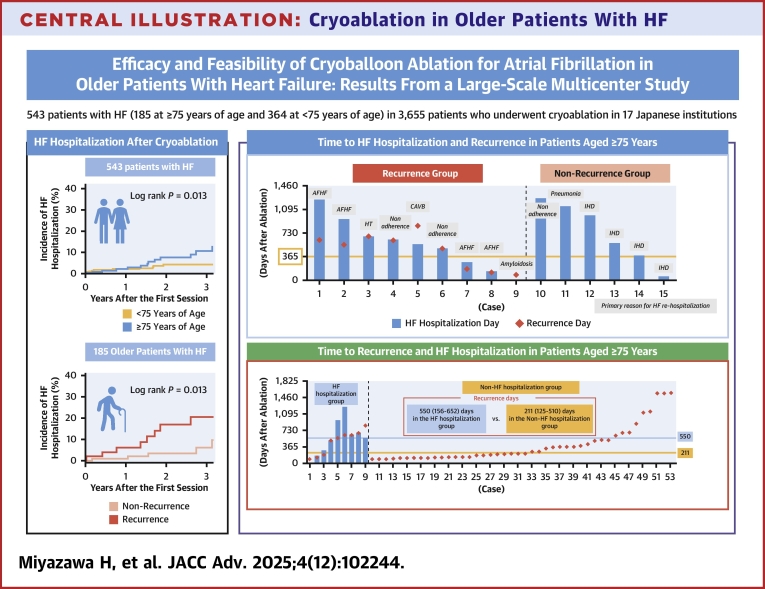
Cryoablation in Older Patients With HF The upper right panel shows the time to HF hospitalization between the recurrence and nonrecurrence groups among the older patients. 73% of HF hospitalizations in older patients occurred after more than 1 year postablation. The gray boxes represent the primary cause of HF hospitalization after ablation. Triggers for worsening HF were AF recurrence, medication nonadherence, discontinuation of hospital visits, and IHD incidence. The lower right panel shows the time to recurrence between patients with and without HF hospitalization among the older patients with recurrence. The median days to recurrence were 550 (156-652) vs 211 (125-510) days in the hospitalization and nonhospitalization groups, respectively. The patients with recurrence and HF hospitalization demonstrated a higher prevalence of CAD and a lower rate of TIC. CAD = coronary artery disease; CAVB = complete atrioventricular block; HT = hypertension; IHD = ischemic heart disease; TIC = tachycardia-induced cardiomyopathy; other abbreviations as in Figure 1.

In addition, most HF hospitalizations occurred >1 year after ablation. In the recurrence group, the triggers of worsening HF did not include only AF recurrence but included factors specific to older adults, such as medication nonadherence and missing hospital visits. In the nonrecurrence group, the primary cause was ischemic heart disease, including myocardial infarction or myocardial ischemia progression, which is generally common in the older population. Details of the triggers of HF hospitalization and baseline etiology in older patients are provided in Table 5. In contrast, in the younger group, multivariable Cox proportional hazards analysis showed that baseline BNP levels, LVEF, structural heart disease, and AF recurrence were independently associated with HF hospitalization after ablation (Supplemental Table 8). Multivariable Cox hazard models with time-dependent covariates and mixed-effects multivariable Cox hazard model for adjusting institutional variability in the older and younger groups are shown in Supplemental Tables 9 and 10.

**Table 5:** Details of the Older Patients Hospitalized for Heart Failure After Ablation

Case	Age(y)	Sex	AF Type	Baseline LVEF	Baseline BNP Levels (pg/dL)	Baseline Etiology	Timing of HF Hospitalization (Months)	Trigger of HF	AF Recurrence	Timing of Recurrence (Months)
1	76	Male	PAF	48.0	229	ICM	41	AF recurrence	Yes	21
2	76	Female	PEF	44.0	860	ICM	32	AF recurrence	Yes	18
3	77	Female	PAF	53.0	38	TIC	23	Hypertension	Yes	23
4	83	Female	PEF	59.0	699	TIC	21	Self-discontinuation of medication	Yes	21
5	80	Female	PAF	58.0	845	TIC	19	Complete atrioventricular block	Yes	28
6	79	Female	PAF	59.0	252	VD	17	Self-discontinuation of medication	Yes	17
7	77	Male	PAF	55.1	782	ICM	9.4	AF recurrence	Yes	6.1
8	77	Female	LSPEF	63.9	173	Other	5.2	AF recurrence	Yes	4.3
9	75	Male	PAF	70.0	73.4	Amyloidosis	0.3	Exacerbation of amyloidosis	Yes	3.0
10	79	Female	PAF	45.5	30	TIC	42	Self-discontinuation of medications	No	
11	81	Female	PAF	46.0	600	TIC	38	Pneumonia	No	
12	85	Female	PAF	58.0	340	TIC	33	Subacute myocardial infarction	No	
13	85	Male	PAF	35.2	1,174	TIC	19	Progression of myocardial ischemia	No	
14	81	Male	PEF	52.1	59	HCM	13	Progression of myocardial ischemia	No	
15	75	Male	PAF	30.3	392	ICM	2.1	Progression of myocardial ischemia	No	

HCM = hypertrophic cardiomyopathy; ICM = ischemic cardiomyopathy; LSPEF = long-standing persistent atrial fibrillation; PAF = paroxysmal atrial fibrillation; PEF = persistent atrial fibrillation; TIC = tachycardia-induced cardiomyopathy; VD = valvular heart disease; other abbreviations as in Table 1.

Details of the triggers of HF hospitalization and baseline etiology in younger patients are provided in Supplemental Table 11. The incidence of triggers for worsening HF, such as recurrence of AF, nonadherence to medication, and ischemic heart disease, was significantly higher in the older group compared to the younger group (27% vs 9.1%, *P* = 0.046; 20% vs 0%, *P* = 0.038; and 27% vs 0%; *P* = 0.013, respectively). Conversely, although the differences were not statistically significant, excessive salt intake and pneumonia were more frequently observed as triggers in the younger group. Among hospitalized patients with HF, the proportion in whom AF recurrence preceded worsening of HF was significantly higher in the older group than in the younger group (47% vs 18%; *P* = 0.009).

For all older patients who experienced AF recurrence after ablation, the time to recurrence, presence or absence of HF hospitalization, and time to HF hospitalization are shown in Central Illustration. Among patients hospitalized for HF, the median time to recurrence was 550 days (approximately 1.5 years after ablation). In contrast, in the non-HF hospitalization group, recurrence occurred within a shorter period, with a median duration of 211 days. Patients with recurrence who were hospitalized for HF demonstrated a higher prevalence of coronary artery disease and a lower rate of TIC than that demonstrated by those with recurrence who were not hospitalized for HF (Supplemental Table 12).

### HF medications after ablation

In the context of drug therapy for HF following ablation, a significantly higher proportion of older patients were prescribed oral diuretics compared to younger ones (42% vs 27%; *P* < 0.001) (Supplemental Table 13), reflecting poorer baseline renal function in the older group (Table 1). Although there were no significant differences between the 2 groups for other oral medications, the use of renin-angiotensin system inhibitors, beta-blockers, and sodium-glucose cotransporter 2 inhibitors tended to be lower in the older group compared to the younger group.

## Discussion

The major findings of this study are as follows: 1) most older patients with HF who underwent catheter ablation for AF had a preserved LVEF with a suspected etiology of TIC; 2) CBA for AF in patients with HF was feasible, showing similar efficacy in recurrence-free survival and mortality rates between the older and younger groups. However, the older group exhibited a significantly higher rate of HF hospitalization than that exhibited by the younger group; 3) cardiac function on echocardiography and BNP levels in the older patients significantly improved postablation, and the effect was more pronounced in the nonrecurrence group than in the recurrence group; 4) high BNP levels and baseline NHYA functional class, but not postablation recurrence, independently associated composite events of death and HF hospitalization in the multivariable analysis; 5) the timing of AF recurrence and HF hospitalization was closely correlated, with most HF hospitalizations occurring more than 1 year after ablation. The triggers for worsening HF were predominantly age-related factors, such as medication nonadherence, discontinuation of hospital attendance, and ischemic heart disease incidence, in addition to recurrence (Central Illustration).

A recent expert consensus guideline recommended that catheter ablation for AF is feasible in patients with concomitant HF.^12^ This recommendation is enhanced by recent randomized studies showing the superiority of radiofrequency catheter ablation over the medical approach in patients with decreased LVEF in terms of mortality and hospitalization for worsening HF.^13^^,^^14^ Cryoablation, a first-option ablation technique, reportedly had significantly better efficacy and safety than that had by radiofrequency ablation, implying its possible usefulness for patients with HF.^15^ However, it is unclear whether older patients aged ≥75 years with HF derive similar benefits from cryoablation like that derived by younger patients. This uncertainty stems from the fact that older patients are often excluded from clinical trials.^5^ Moreover, even in studies that include patients aged ≥75 years,^16^^,^^17^ the sample size was typically small, and they did not focus on individuals with HF specifically. Consequently, key prognostic outcomes such as postoperative hospitalization for HF or mortality have not been adequately examined. This study is the first to evaluate outcomes after cryoablation in a larger cohort of older patients with HF and to include a follow-up period exceeding approximately 2 years, which provides valuable insights, especially given the lack of relevant studies in this area. Additionally, we did not only analyze AF recurrence but also outcomes such as changes in cardiac function and BNP levels, hospitalization for HF, and mortality, which are typically assessed as essential endpoints in patients with HF. Outcomes, such as recurrence and mortality, were similar between the older and younger patients; however, HF hospitalization significantly occurred more often in the older group than in the younger group. Furthermore, older patients with recurrent AF had significantly higher rates of HF hospitalization and cardiovascular mortality than those without recurrence. This trend may be attributed to the comparatively smaller improvements in LVEF and BNP levels observed in the recurrence subgroup than in the nonrecurrence subgroup.

Previous studies on cryoablation in older patients have primarily focused on AF recurrence as the main outcome.^16^^,^^17^ However, detailed reports examining postoperative hospitalization for HF are lacking. Once HF occurs, the condition gradually worsens, even after recovery from HF exacerbation, resulting in a malignant cycle that leads to poor prognosis.^18^ Most HF develops due to AF occurrence, and up to 70% of the older population may have the etiology of AF-related HF. Therefore, suggesting that AF recurrence after ablation could be directly linked to worsening HF, leading to subsequent rehospitalization for HF, seems reasonable. The results of our study demonstrated that the timing of AF recurrence was closely associated with HF hospitalization in the older group, with most hospitalizations occurring more than 1 year after ablation, supporting the abovementioned hypothesis. Although follow-up postablation is often concluded if there is no recurrence within 1 year of hospital visits,^1^ it is recommended that the follow-up period for older patients should be >1 year and conducted with greater caution, necessitating shorter intervals between visits compared to that observed in younger patients. Moreover, nonadherence to medications and unexpected interruptions in hospital visits are commonly observed among older people, and many comorbid conditions, cognitive dysfunction, and vision or hearing impairment may be attributed to the situations of older adults.^19^ In our study, the higher prevalence of postmedication of diuretics in the older group might indicate that among such older patients who are dependent on diuretics, nonadherence to medication may be a major contributing factor to the exacerbation of HF. Even though cognitive and physical dysfunction might not be apparent at the time of the first cryoablation, these impairments could emerge in the long-term after the ablation. Therefore, special observations of the older population are increasingly needed because they still live their lives after systematic postablation follow-up. In contrast, the progression of ischemic heart disease is also frequently observed in older patients and is the primary trigger for worsening HF in the nonrecurrence group. Comprehensive care, encompassing not only monitoring for long-term recurrence but also ensuring medication adherence, maintaining regular hospital visits, obtaining help from family or caregivers, and managing cardiac complications beyond arrhythmia, is crucial for older patients.

HF with preserved ejection fraction (HFpEF) is common among older patients, affecting >10% of those in their 80s.^20^ Approximately half of these patients also have AF. However, the efficacy of catheter ablation has been primarily demonstrated in patients with reduced LVEF, and evidence regarding HFpEF remains scarce. In particular, older patients with HFpEF have often been excluded from clinical trials, and drug therapy has remained the standard treatment. Our study demonstrated that the proportion of HFpEF among older patients with HF was notably high (78%), with the efficacy of CBA being notable even in this population. The CABA-HFPEF-DZHK27 (CAtheter-Based Ablation of atrial fibrillation compared to conventional treatment in patients with Heart Failure with Preserved Ejection Fraction; NCT05508256) trial—the first large-scale randomized controlled trial designed to prospectively assess the efficacy of catheter ablation in patients with HFpEF and AF—is currently ongoing,^20^ and the results from this trial may further support our findings.

In our study, the mean CHA_2_DS_2_-VASc score among older patients with coexisting AF and HF was high at 4.7 ± 1.1, with 162 (88%) of them having a CHA_2_DS_2_-VASc score of ≥4. The median duration of AF was 0.5 years, with 125 (68%) older patients undergoing CBA within 1 year of the diagnosis. In patients with AF having CHA_2_DS_2_-VASc scores of ≥4 and multiple comorbidities, early rhythm control therapy has been shown to significantly reduce the incidence of the primary adverse cardiovascular outcomes of cardiovascular death, stroke, worsening HF, and acute coronary syndrome in a subanalysis of EAST-AFNET4 (Early Treatment of Atrial Fibrillation for Stroke Prevention Trial–Atrial Fibrillation Network).^21^ A future prospective, randomized study (EASThigh-AFNET 11 [Early Atrial Fibrillation Ablation for Stroke Prevention in Patients with High Comorbidity Burden] trial; NCT06324188) will verify the effectiveness of early ablation therapy in patients with AF and HF who have multiple comorbidities.

Furthermore, recent advances in ablation technology, such as pulsed field ablation, have progressively increased as they are readily available with a potential application in patients with HF.^22^^,^^23^ A recent substudy of the MANIFEST-PF (Multi-National Survey on the Methods, Efficacy, and Safety on the Post-Approval Clinical Use of Pulsed Field Ablation) trial demonstrated that pulsed field ablation was feasible, with a 1-year recurrence-free rate of 67.5% to 71.3% among 207 patients with AF concomitant with HF.^22^ However, a comparative study with different ablation energies is warranted to seek the best approach for the older population from the viewpoints of safety, effectiveness, and other prognostic outcomes.

### Study limitations

This retrospective study conducted in Japan primarily included older patients with preserved or mildly reduced LVEF. Future research on CBA in older patients with reduced LVEF (≤40%) is needed. Some patients lacked follow-up data, such as BNP levels or echocardiographic findings. Variations in follow-up schedules and treatments at each institution may have influenced the outcomes. However, the 1-year recurrence-free rate was comparable to that reported in previous studies,^16^^,^^17^ indicating that our follow-up protocols may not be unusual. This study did not assess symptom recovery, NYHA functional class, or quality of life after the follow-up period. The CIs were not adjusted for multiple comparisons and should be interpreted with caution. Patients with HF were stratified by preprocedure LVEF on echocardiography, which might have changed with the recent onset and management of HF. Moreover, the timing of BNP levels and echocardiography assessments varied during the follow-up, and these tests may have mostly been performed during emergency visits for unusual cases, potentially skewing the results in the event group. This study did not evaluate frailty which is often observed in older patients and is associated with worse outcomes in patients with HF undergoing catheter ablation for AF.^24^ Medication adherence was not systematically collected in all patients in this study. Finally, the etiology of HF was determined by each institution without the systematic use of advanced imaging or biopsies in all patients. In particular, although >70% of patients in this study were diagnosed with HFpEF (Heart Failure Association-Pre-test assessment, Echocardiography and natriuretic peptide score, Functional testing in cases of uncertainty, Final aetiology), the HFA-PEFF score, known for its high reproducibility and objectivity, was not used.^25^ In addition, we did not determine a universal definition for TIC (eg, the cutoff for LVEF recovery on echocardiography) in advance.

## Conclusions

This large multicenter study demonstrated that CBA for AF in older patients with HF was both effective and feasible, comparable to the outcomes in younger patients, with improvements in cardiac function and favorable prognosis. For older adults, providing special care and a tailored approach is essential for follow-up postablation.

Perspectives **COMPETENCY IN MEDICAL KNOWLEDGE:** CBA for AF in HF patients was feasible, with similar recurrence-free survival and mortality rates across age groups, but a significantly higher HF hospitalization rate in older patients. For older patients with HF undergoing CBA for AF, a follow-up period of over 1 year with shorter intervals between visits may be helpful and could include monitoring for recurrence, medication adherence, regular hospital visits, family or caregiver support, and management of cardiac complications beyond arrhythmia.

**TRANSLATIONAL OUTLOOK:** Recent advances in ablation technology, such as pulsed field ablation, have expanded for potential use in patients with HF, but further comparative studies on different ablation energies are needed to determine the safest and most effective approach for older patients.

## Funding support and author disclosures

Drs Yanagisawa and Shibata are affiliated to a department sponsored by 10.13039/100019341Medtronic, Japan. All other authors have reported that they have no relationships relevant to the contents of this paper to disclose.
