# Supplementary material for: Cryoballoon Ablation in Older Patients With Heart Failure: Insights From a Large-Scale Multicenter Clinical Study
Source: JACC Adv. 2025 Oct 29;4(12):102244. doi: 10.1016/j.jacadv.2025.102244 (PMC12805183; doi:10.1016/j.jacadv.2025.102244)
Supplement: Supplemental Tables 1 to 13 and Figures 1 to 5 [file mmc1.docx]

**Supplemental Materials**

Miyazawa *et al.*: Cryoballoon ablation in older patients with heart failure: Insights from a large-scale multicenter clinical study

**Table of contents**

**Page 3:** *Supplemental Table 1.* Factors associated with composite events of all-cause death and HF hospitalization after cryoballoon ablation in older patients with HF in a multivariable Cox hazard model with time-dependent covariates

**Page 4:** *Supplemental Table 2.* Factors associated with composite events of all-cause death and HF hospitalization in older patients in mixed-effect multivariable Cox hazard model

**Page 5:** *Supplemental Table 3.* Characteristics of four patients with NHYA class III/IV before cryoablation

**Page 6–7:** *Supplemental Table 4.* Comparison of baseline characteristics among the older groups according to baseline LVEF

**Page 8–9:** *Supplemental Table 5.* Comparison of baseline characteristics among the older groups according to NYHA class

**Page 10:** *Supplemental Table 6.* Changes in BNP levels and LVEF post-ablation in older patients with HF

**Page 11–12:** *Supplemental Table 7.* Factors associated with HF hospitalization after cryoballoon ablation in older patients with HF

**Page 13–14:** *Supplemental Table 8.* Factors associated with HF hospitalization after cryoballoon ablation in patients with HF aged <75 years

**Page 15:** *Supplemental Table 9.* Factors associated with HF hospitalization after cryoballoon ablation in older and younger groups in a time-dependent multivariable Cox hazard model with time-dependent covariates

**Page 16:** *Supplemental Table 10.* Factors associated with HF hospitalization after ablation in older and younger groups in mixed-effect multivariable Cox hazard model

**Page 17:** *Supplemental Table 11.* Details of patients aged <75 years hospitalized for HF after ablation

**Page 18–19:** *Supplemental Table 12.* Comparison of baseline characteristics in older patients with AF recurrence

**Page 20:** *Supplemental Table 13.* Comparison of post-ablation HF medication use between older and younger patients

**Page 21:** *Supplemental Figure 1.* Survival curves using the Simon-Makuch analysis after adjusting for a time-dependent event, recurrence factor between patients with HF aged ≥75 or <75 years

**Page 22:** *Supplemental Figure 2.* Estimated risk of outcomes after cryoablation according to age, accounting for the competing risk of death

**Page 23:** *Supplemental Figure 3.* Survival curves using Simon-Makuch analysis after adjusting for a time-dependent event; recurrence factor in older patients with or without recurrence

**Page 24:** *Supplemental Figure 4.* Kaplan–Meier survival curves of AF recurrence (A), all-cause death (B), cardiovascular death (C), and HF hospitalization (D) between patients with PAF and PEF

**Page 25:** *Supplemental Figure 5.* Survival curves using Simon-Makuch analysis after the adjustment of a time-dependent event, recurrence factor between older patients with PAF and PEF.

**Page 26:** *Reference*

**Supplemental Table 1.** Factors associated with composite events of all-cause death and HF hospitalization after cryoballoon ablation in older patients with HF in a multivariable Cox hazard model with time-dependent covariates

| Parameters | Multivariable analysis | | |
| --- | --- | --- | --- |
|  | HR (95% CI) | *p* value |  |
| Creatinine clearance level, mL/min | 0.975 (0.946–1.006) | 0.112 |  |
| Ln BNP, pg/dL | 1.851 (0.855–4.011) | <0.018 |  |
| NYHA class | 1.746 (0.976–3.121) | 0.060 |  |

Data from 179 patients were analyzed. Four predefined variables (Creatinine clearance level, Ln BNP, NYHA class, and recurrence) that could be associated with the events were included in the multivariable model. The recurrence factor was included as a time-dependent variable. Model assumptions were evaluated using the Akaike's Information Criterion (AIC) minimization method,^1^ and the most probable combination of factors was selected. BNP levels were logarithmically transformed due to the wide range of distributions. BNP, B-type natriuretic peptide; CI, confidence interval; HF, heart failure; HR, hazard ratio; NYHA, New York Heart Association.

**Supplemental Table 2.** Factors associated with composite events of all-cause death and HF hospitalization in older patients in mixed-effect multivariable Cox hazard model

| Parameters | Multivariable analysis | | |
| --- | --- | --- | --- |
|  | HR (95% CI) | *p* value |  |
| NYHA class | 2.441 (1.160–5.136) | 0.019 |  |
| Ln BNP, pg/dL | 1.890 (1.123–3.179) | 0.016 |  |
| Recurrence | 1.769 (0.740–4.229) | 0.199 |  |

Data from 179 patients were analyzed. Four predefined variables (Creatinine clearance level, Ln BNP, NYHA class, and recurrence) that could be associated with the events were included in the multivariable model. The model assumption was assessed using the Akaike's Information Criterion (AIC) minimization method,^1^ and the combination of factors with the highest probability was selected. BNP levels were logarithmically transformed due to their wide range of distributions. BNP, B-type natriuretic peptide; CI, confidence interval; HF, heart failure; HR, hazard ratio; NYHA, New York Heart Association.

**Supplemental Table 3.** Characteristics of four patients with NHYA class III/IV before cryoablation

| Case | Age, years | Sex | Type of AF | Duration of AF, years | BMI, kg/m^2^ | NYHA class | BNP levels, pg/dL | LAD, mm | LVEF, % | CHADS_2_ score | CHA_2_DS_2_-VASc score | Etiology of HF | Outcomes | Days of outcomes |
| --- | --- | --- | --- | --- | --- | --- | --- | --- | --- | --- | --- | --- | --- | --- |
| 1 | 76 | Female | PEF | 1 | 24.2 | III | 859.6 | 40 | 44 | 4 | 7 | IHD | Recurrence, HF hospitalization | 550, 952 |
| 2 | 79 | Male | PAF | 2 | 27.9 | III | 30.3 | 52 | 64 | 6 | 7 | TIC | - | - |
| 3 | 81 | Female | PAF | 4 | 23.9 | III | 52.9 | 40.8 | 69.2 | 2 | 3 | VD | Non-CVD | 720 |
| 4 | 81 | Male | PEF | 2 | 19.7 | III | 13.6 | 43.9 | 60.1 | 5 | 5 | TIC | Recurrence | 116 |

AF, atrial fibrillation; BMI, body mass index; BNP, B-type natriuretic peptide; CVD, cardiovascular death; HF, heart failure; IHD, ischemic cardiomyopathy; LAD, left atrial diameter; LVEF, left ventricular ejection fraction; NYHA, New York Heart Association; PAF, paroxysmal AF; PEF, persistent AF; TIC, tachycardia-induced cardiomyopathy; VD, valvular heart disease.

**Supplemental Table 4.** Comparison of baseline characteristics among the older groups according to baseline LVEF

|  | LVEF ≤40%  (n=15) | LVEF 40–49%  (n=25) | LVEF ≥50%  (n=145) | *p*-value |
| --- | --- | --- | --- | --- |
| Age, years | 77.9±3.4 | 78.5±2.9 | 79.3±3.8 | 0.277 |
| Male sex | 13 (87%) | 11 (44%) | 54 (37%) | 0.001 |
| Body weight, kg | 57.5±9.0 | 55.1±7.8 | 56.1±10.6 | 0.771 |
| Body mass index, kg/m^2^ | 21.1±2.6 | 22.6±2.4 | 23.1±3.6 | 0.035 |
| Duration of AF, years | 0.5 (0.4–1.0) | 0.5 (0.3–1.0) | 0.5 (0.3–2.0) | 0.989 |
| AF type |  |  |  |  |
| Paroxysmal | 8 (53%) | 12 (48%) | 114 (79%) | 0.002 |
| Persistent | 7 (47%) | 13 (52%) | 31 (21%) | 0.002 |
| Long-standing persistent | 0 (0%) | 1 (4.0%) | 3 (2.1%) | 0.626 |
| Antiarrhythmic drugs |  |  |  |  |
| Class I | 4 (27%) | 3 (12%) | 37 (26%) | 0.329 |
| Class III | 3 (20%) | 4 (16%) | 19 (13%) | 0.638 |
| NYHA class | 1.7±0.5 | 1.5±0.6 | 1.3±0.5 | 0.045 |
| Comorbidity |  |  |  |  |
| Hypertension | 8 (53%) | 16 (64%) | 109 (75%) | 0.129 |
| Diabetes mellitus | 3 (20%) | 9 (36%) | 27 (19%) | 0.144 |
| Coronary artery disease | 1 (6.7%) | 8 (32%) | 9 (6.2%) | 0.002 |
| Stroke/TIA | 2 (13%) | 2 (8.0%) | 18 (12%) | 0.844 |
| Hemodialysis | 0 (0%) | 0 (0%) | 3 (2.1%) | NA |
| Echocardiographic data |  |  |  |  |
| LAD, mm | 42.8±6.3 | 40.9±6.6 | 39.8±6.0 | 0.158 |
| LVEDD, mm | 52.3±9.8 | 47.9±6.7 | 45.1±5.6 | 0.011 |
| LVEDS, mm | 45.1±10.1 | 36.7±6.1 | 29.5±4.4 | <0.001 |
| LVEF, % | 30.8±8.1 | 45.3±2.6 | 63.2±5.6 | <0.001 |
| CHADS_2_ score | 2.9±1.1 | 3.1±1.1 | 3.1±1.0 | 0.725 |
| CHA_2_DS_2_-VASc score | 4.2±1.0 | 4.8±1.2 | 4.7±1.1 | 0.167 |
| Laboratory data |  |  |  |  |
| Creatinine clearance, mL/min | 43.2±14.4 | 47.2±13.6 | 48.0±14.6 | 0.466 |
| BNP levels, pg/dL | 333 (134–629) | 270 (156–402) | 155 (117–251) | <0.001 |
| DOAC | 12 (80%) | 24 (96%) | 127 (88%) | 0.237 |
| History of device implantation |  |  |  |  |
| Pacemaker | 1 (6.7%) | 2 (8.0%) | 10 (6.9%) | 0.878 |
| ICD | 0 (0%) | 2 (8.0%) | 0 (0%) | 0.024 |
| CRT | 1 (6.7%) | 0 (0%) | 1 (0.7%) | 0.174 |
| Medications |  |  |  |  |
| ACEI/ARB | 7 (47%) | 18 (72%) | 68 (47%) | 0.065 |
| ARNI | 0 (0%) | 0 (0%) | 0 (0%) | NA |
| Beta-blocker | 13 (87%) | 22 (88%) | 80 (55%) | 0.001 |
| Spironolactone | 5 (33%) | 9 (36%) | 25 (17%) | 0.050 |
| SGLT-2 inhibitor | 1 (6.7%) | 3 (12%) | 2 (1.4%) | 0.025 |
| Diuretic | 7 (47%) | 16 (64%) | 59 (41%) | 0.094 |
| Etiology of HF |  |  |  | 0.481 |
| Tachycardia-induced cardiomyopathy | 9 (60%) | 18 (72%) | 102 (70%) |  |
| Ischemic cardiomyopathy | 2 (13%) | 6 (24%) | 19 (13%) |  |
| Dilated cardiomyopathy | 3 (20%) | 0 (0%) | 0 (0%) |  |
| Hypertrophic cardiomyopathy | 0 (0%) | 0 (0%) | 5 (3.4%) |  |
| Valvular heart disease | 0 (0%) | 0 (0%) | 13 (9.0%) |  |
| Sarcoidosis | 0 (0%) | 0 (0%) | 0 (0%) |  |
| Amyloidosis | 0 (0%) | 0 (0%) | 1 (0.7%) |  |
| Other | 1 (6.7%) | 1 (4.0%) | 5 (3.4%) |  |
| Session time, min (from puncture to session end) | 136±53 | 131±47 | 133±38 | 0.919 |
| Major complications | 1 (6.7%) | 0 (0%) | 9 (6.2%) | 0.481 |

ACEI, angiotensin-converting enzyme inhibitor; AF, atrial fibrillation; ARB, angiotensin receptor blocker; ; ARNI, angiotensin receptor neprilysin inhibitor; BNP, B-type natriuretic peptide; CRT, cardiac resynchronization therapy; DOAC, direct oral anticoagulant; HF, heart failure; ICD, implantable cardioverter-defibrillator; LAD, left atrial diameter; LVEDD, left ventricular end-diastolic diameter; LVESD, left ventricular end-systolic diameter; LVEF, left ventricular ejection fraction; NYHA, New York Heart Association; SGLT, sodium glucose co-transporter; TIA, transient ischemic attack.

**Supplemental Table 5.** Comparison of baseline characteristics among the older groups according to NYHA class

|  | NYHA class I (n=118) | NYHA class II (n=63) | NYHA class III–IV (n=4) | *p* value |
| --- | --- | --- | --- | --- |
| Age, years | 79.2 ± 3.8 | 78.9 ± 3.4 | 79.3 ± 2.4 | 0.872 |
| Male sex | 49 (42%) | 27 (43%) | 2 (50%) | 0.955 |
| Body weight, kg | 55.8 ± 9.5 | 56.4 ± 11.2 | 59.0 ± 12.1 | 0.767 |
| Body mass index, kg/m^2^ | 22.9 ± 3.2 | 22.8 ± 3.9 | 23.9 ± 3.4 | 0.822 |
| Duration of AF, years | 0.5 (0.2–2.0) | 0.6 (0.3–1.9) | 2.0 (1.3–3.5) | 0.103 |
| AF type |  |  |  |  |
| Paroxysmal | 94 (80%) | 38 (60%) | 2 (50%) | 0.013 |
| Persistent | 24 (20%) | 25 (40%) | 2 (50%) | 0.013 |
| Long-standing persistent | 1 (0.8%) | 3 (4.8%) | 0 (0%) | 0.196 |
| Antiarrhythmic drugs |  |  |  |  |
| Class I | 32 (27%) | 12 (19%) | 0 (0%) | 0.310 |
| Class III | 10 (8.5%) | 13 (21%) | 3 (75%) | 0.001 |
| Comorbidity |  |  |  |  |
| Hypertension | 85 (72%) | 45 (71%) | 3 (75%) | NA |
| Diabetes mellitus | 24 (20%) | 13 (21%) | 2 (50%) | 0.370 |
| Coronary artery disease | 9 (7.6%) | 8 (13%) | 1 (25%) | 0.177 |
| Stroke/TIA | 15 (13%) | 5 (7.9%) | 2 (50%) | 0.061 |
| Hemodialysis | 2 (1.7%) | 1 (1.6%) | 0 (0%) | NA |
| Echocardiographic data |  |  |  |  |
| LAD, mm | 39.4 ± 6.2 | 41.4 ± 5.8 | 44.1 ± 5.3 | 0.042 |
| LVEDD, mm | 45.4 ± 6.1 | 47.2 ± 6.9 | 48.9 ± 7.1 | 0.146 |
| LVEDS, mm | 30.6 ± 5.4 | 33.6 ± 9.1 | 34.0 ± 8.8 | 0.117 |
| LVEF, % | 60.0 ± 9.5 | 54.6 ± 14.1 | 59.3 ± 10.8 | 0.084 |
| CHADS_2_ score | 3.0 ± 1.0 | 3.0 ± 0.8 | 4.3 ± 1.7 | 0.047 |
| CHA_2_DS_2_-VASc score | 4.6 ± 1.1 | 4.7 ± 0.9 | 5.5 ± 1.9 | 0.599 |
| Laboratory data |  |  |  |  |
| Creatinine clearance, mL/min | 49.6 ± 12.9 | 44.2 ± 16.2 | 36.2 ± 16.9 | 0.087 |
| BNP levels, pg/dL | 157(120–262) | 232(127–356) | 42 (18–658) | 0.013 |
| DOAC | 109 (92%) | 50 (79%) | 4 (100%) | 0.033 |
| History of device implantation |  |  |  |  |
| Pacemaker | 8 (6.8%) | 5 (7.9%) | 0 (0%) | 0.828 |
| ICD | 1 (0.8%) | 0 (0%) | 1 (25%) | 0.043 |
| CRT | 0 (0%) | 2 (3.2%) | 0 (0%) | 0.158 |
| Medications |  |  |  |  |
| ACEI/ARB | 54 (46%) | 38 (60%) | 1 (25%) | 0.100 |
| ARNI | 0 (0%) | 0 (0%) | 0 (0%) | NA |
| Beta-blocker | 63 (53%) | 49 (78%) | 3 (75%) | 0.003 |
| Spironolactone | 16 (14%) | 20 (32%) | 3 (75%) | 0.001 |
| SGLT-2 inhibitor | 6 (3.2%) | 4 (6.3%) | 1 (25%) | 0.012 |
| Diuretic | 30 (25%) | 48 (76%) | 4 (100%) | <0.001 |
| Etiology of HF |  |  |  | 0.750 |
| Tachycardia-induced cardiomyopathy | 83 (70%) | 44 (70%) | 2 (50%) |  |
| Ischemic cardiomyopathy | 22 (19%) | 4 (6.3%) | 1 (25%) |  |
| Dilated cardiomyopathy | 1 (0.8%) | 2 (3.2%) | 0 (0%) |  |
| Hypertrophic cardiomyopathy | 3 (2.5%) | 2 (3.2%) | 0 (0%) |  |
| Valvular heart disease | 6 (5.1%) | 6 (9.5%) | 1 (25%) |  |
| Sarcoidosis | 0 (0%) | 0 (0%) | 0 (0%) |  |
| Amyloidosis | 0 (0%) | 1 (1.6%) | 0 (0%) |  |
| Other | 3 (2.5%) | 4 (6.3%) | 0 (0%) |  |
| Session time, min (from puncture to session end) | 126 ± 32 | 143 ± 51 | 166 ± 28 | 0.013 |
| Major complications | 7 (5.9%) | 3 (4.8%) | 0 (0%) | NA |

Abbreviations are as in Supplemental Table 4.

**Supplemental Table 6.** Changes in BNP levels and LVEF post-ablation in older patients with HF

| Group | Baseline BNP level | Follow-up BNP level | *p*-value |
| --- | --- | --- | --- |
| LVEF ≤40% (n=15) | 333 (134–630) | 90 (39–561) | 0.070 |
| LVEF 40–49% (n=25) | 270 (156–402) | 111 (40–308) | 0.024 |
| LVEF ≥50% (n=145) | 155 (117–251) | 103 (54–188) | <0.001 |
| NYHA class I (n=118) | 157 (120–262) | 81 (37–199) | <0.001 |
| NYHA class II (n=63) | 232 (127–356) | 124 (73–234) | 0.002 |
| NYHA class III–IV (n=4) | 42 (18–658) | 118 (30–274) | 0.210 |
| Group | LVEF baseline | LVEF follow-up |  |
| LVEF ≤40% (n=15) | 31.4 ± 7.0 | 52.8 ± 14.1 | 0.567 |
| LVEF 40–49% (n=25) | 45.4 ± 2.5 | 50.8 ± 12.5 | 0.369 |
| LVEF ≥50% (n=145) | 62.6 ± 5.2 | 62.6 ± 8.2 | <0.001 |
| NYHA class I (n=118) | 59.2 ± 9.5 | 61.0 ± 9.5 | <0.001 |
| NYHA class II (n=63) | 54.0 ± 13.6 | 59.0 ± 11.4 | 0.002 |
| NYHA class III–IV (n=4) | 57.8 ± 12.8 | 47.3 ± 21.2 | N/A |

Regarding the association between BNP levels and echocardiographic data before and after treatment, we conducted a linear regression analysis using post-treatment value as the outcome variable, and pre-treatment value and the measurement interval as covariates, taking into account the duration between measurements. BNP levels were logarithmically transformed for analysis. BNP, B-type natriuretic peptide; HF, heart failure; LVEF, left ventricular ejection fraction; NYHA, New York Heart Association.

**Supplemental T****able 7.** Factors associated with HF hospitalization after cryoballoon ablation in older patients with HF

| Parameters | Univariable analysis | | Multivariable analysis | |
| --- | --- | --- | --- | --- |
|  | HR (95% CI) | *p* value | HR (95% CI) | *p* value |
| Age, years | 0.996 (0.862–1.151) | 0.958 |  |  |
| Female sex | 0.953 (0.339–2.681) | 0.927 |  |  |
| Body mass index, kg/m^2^ | 1.062 (0.928–1.215) | 0.384 |  |  |
| AF duration, years | 1.057 (0.921–1.214) | 0.429 |  |  |
| Creatinine clearance level, mL/min | 0.974 (0.941–1.008) | 0.128 |  |  |
| BNP, pg/dL | 1.003 (1.001–1.004) | <0.001* |  |  |
| LAD, mm | 1.055 (0.971–1.147) | 0.203 |  |  |
| LVEF, % | 0.958 (0.926–0.991) | 0.014* | 0.953 (0.912–0.996) | 0.032 |
| LVEDD, mm | 1.091 (1.024–1.164) | 0.008* |  |  |
| CHADS_2_ score | 1.544 (0.982–2.428) | 0.060 |  |  |
| CHA₂DS₂-VASc score | 1.666 (1.054–2.633) | 0.029* | 1.487 (0.931–2.377) | 0.097 |
| Persistent AF | 0.950 (0.302–2.986) | 0.930 |  |  |
| NYHA class | 3.299 (1.450–7.508) | 0.004* | 2.147 (0.937–4.921) | 0.071 |
| Structural heart disease | 2.211 (0.801–6.106) | 0.126 |  |  |
| Recurrence | 3.106 (1.104–8.737) | 0.032* | 3.587 (1.194–10.78) | 0.023 |
| Early recurrence | 0.721 (0.203–2.556) | 0.612 |  |  |
| History of device implantation | 2.238 (0.629–7.961) | 0.213 |  |  |

Data from 182 patients were analyzed. *Variables included in the multivariable model using the backward stepwise method. AF, atrial fibrillation; BNP, B-type natriuretic peptide; CI, confidence interval; HF, heart failure; HR, hazard ratio; LAD, left atrial diameter; LVEDD, left ventricular end-diastolic diameter; LVEF, left ventricular ejection fraction; NYHA, New York Heart Association.

**Supplemental Table 8.** Factors associated with HF hospitalization after cryoballoon ablation in patients with HF aged <75 years

| Parameters | Univariable analysis | | Multivariable analysis | |
| --- | --- | --- | --- | --- |
|  | HR (95% CI) | *p* value | HR (95% CI) | *p* value |
| Age, years | 0.965 (0.905–1.028) | 0.266 |  |  |
| Female sex | 0.700 (0.186–2.640) | 0.599 |  |  |
| Body mass index, kg/m^2^ | 1.022 (0.889–1.175) | 0.756 |  |  |
| AF duration, years | 0.906 (0.676–1.213) | 0.507 |  |  |
| Creatinine clearance level, mL/min | 0.994 (0.972–1.015) | 0.557 |  |  |
| BNP, pg/dL | 1.002 (1.001–1.004) | <0.001* | 1.002 (1.000–1.004) | 0.014 |
| LAD, mm | 1.160 (1.067–1.260) | 0.001* |  |  |
| LVEF, % | 0.921 (0.886–0.957) | <0.001* | 0.953 (0.915–0.992) | 0.019 |
| LVEDD, mm | 1.037 (1.004–1.071) | 0.026* |  |  |
| CHADS_2_ score | 1.025 (0.569–1.848) | 0.934 |  |  |
| CHA₂DS₂-VASc score | 1.034 (0.666–1.606) | 0.881 |  |  |
| Persistent AF | 0.541 (0.117–2.506) | 0.433 |  |  |
| NYHA class | 2.478 (1.299–4.726) | 0.006* |  |  |
| Structural heart disease | 7.035 (1.514–32.69) | 0.013* | 6.612 (1.148–38.07) | 0.034 |
| Recurrence | 4.634 (1.186–18.11) | 0.027* | 4.564 (1.160–17.96) | 0.030 |
| Early recurrence | 2.175 (0.660–7.164) | 0.201 |  |  |
| History of device implantation | 1.100 (0.140–8.648) | 0.928 |  |  |

Data from 359 patients were analyzed. *Variables included in the multivariable model using the backward stepwise method. AF, atrial fibrillation; BNP, B-type natriuretic peptide; CI, confidence interval; HF, heart failure; HR, hazard ratio; LAD, left atrial diameter; LVEDD, left ventricular end-diastolic diameter; LVEF, left ventricular ejection fraction; NYHA, New York Heart Association.

**Supplemental Table 9.** Factors associated with HF hospitalization after cryoballoon ablation in older and younger groups in a time-dependent multivariable Cox hazard model with time-dependent covariates

| Older group | Multivariable analysis | | |
| --- | --- | --- | --- |
|  | HR (95% CI) | *p* value |  |
| NYHA class | 2.464 (0.938–6.472) | 0.067 |  |
| Ln BNP, pg/dL | 0.956 (0.919–0.994) | <0.018 |  |
| Recurrence | 2.704 (0.755–9.679) | 0.126 |  |
| Younger group |  |  |  |
| NYHA class | 2.517 (0.960–6.597) | 0.060 |  |
| Ln BNP, pg/dL | 0.932 (0.874–0.994) | 0.033 |  |

Data from 179 and 348 patients were analyzed in the older and younger groups, respectively. Four predefined variables (left ventricular ejection fraction, Ln BNP, NYHA class, and recurrence) that could be associated with the events were included in the multivariable model. The recurrence factor was included as a time-dependent variable. Model assumptions were evaluated using the Akaike's Information Criterion (AIC) minimization method,^1^ and the most probable combination of factors was selected. BNP levels were logarithmically transformed due to the wide range of distributions. BNP, B-type natriuretic peptide; CI, confidence interval; HF, heart failure; HR, hazard ratio; NYHA, New York Heart Association.

**Supplemental Table 10.** Factors associated with HF hospitalization after ablation in older and younger groups in mixed-effect multivariable Cox hazard model

| Older group | Multivariable analysis | | |
| --- | --- | --- | --- |
|  | HR (95% CI) | *p* value |  |
| NYHA class | 3.534 (1.625–7.686) | 0.001 |  |
| Recurrence | 3.789 (1.340–10.715) | 0.012 |  |
| Younger group |  |  |  |
| Ln BNP, pg/dL | 2.763 (1.267–6.025) | 0.011 |  |
| NYHA class | 2.342 (1.019–5.383) | 0.045 |  |
| Recurrence | 6.407 (1.432–28.664) | 0.033 |  |

Data from 179 and 348 patients were analyzed in the older and younger groups, respectively. Four predefined variables (left ventricular ejection fraction, Ln BNP, NYHA class, and recurrence) that could be associated with the events were included in the multivariable model. The recurrence factor was included as a time-dependent variable. Model assumptions were evaluated using the Akaike's Information Criterion (AIC) minimization method,^1^ and the most probable combination of factors was selected. BNP levels were logarithmically transformed due to the wide range of distributions. BNP, B-type natriuretic peptide; CI, confidence interval; HF, heart failure; HR, hazard ratio; NYHA, New York Heart Association.

**Supplemental Table 11.** Details of patients aged <75 years hospitalized for HF after ablation

| Case | Age (years) | Sex | AF type | Baseline  LVEF | Baseline  BNP levels  (pg/dL) | Baseline etiology | Timing of HF hospitalization (months) | Trigger of HF | AF recurrence | Timing of recurrence (months) |
| --- | --- | --- | --- | --- | --- | --- | --- | --- | --- | --- |
| 1 | 69 | Male | PAF | 61 | 352.1 | TIC | 51 | Urinary tract infection | Yes | 3.3 |
| 2 | 62 | Male | PAF | 15 | 678.5 | DCM | 24 | Progression of DCM | Yes | 25 |
| 3 | 67 | Male | PAF | 24 | 309 | DCM | 21 | Excessive salt intake | No |  |
| 4 | 57 | Female | PAF | 36 | 117.3 | HCM | 20 | Excessive salt intake | Yes | 8.3 |
| 5 | 57 | Male | PAF | 18 | 1455.7 | TIC | 16 | Hypertension | No |  |
| 6 | 70 | Male | PAF | 47 | 209 | ICM | 8.3 | Influenza infection | No |  |
| 7 | 59 | Female | PAF | 59 | 206.1 | Other | 2.6 | Atrial tachycardia | Yes | 3.1 |
| 8 | 74 | Male | PEF | 24 | 901.7 | ICM | 2.2 | Pneumonia | Yes | 6.7 |
| 9 | 67 | Female | PAF | 31 | 493.5 | ICM | 1.1 | Pneumonia | No |  |
| 10 | 54 | Male | PEF | 35 | 215.8 | DCM | 0.7 | Early AF recurrence | Yes | 34 |
| 11 | 48 | Male | PAF | 63 | 102 | HCM | 0.1 | Pneumonia | Yes | 11 |

AF, atrial fibrillation; BNP, B-type natriuretic peptide; DCM, dilated cardiomyopathy; HCM, hypertrophic cardiomyopathy; HF, heart failure; ICM, ischemic cardiomyopathy; LVEF, left ventricular ejection fraction; PAF, paroxysmal atrial fibrillation; PEF, persistent atrial fibrillation; TIC, tachycardia-induced cardiomyopathy.

**Supplemental Table 12.** Comparison of baseline characteristics in older patients with AF recurrence

|  | HF hospitalization (n=9) | Non-HF hospitalization (n=44) | *p* value |
| --- | --- | --- | --- |
| Age, years | 77.8±2.5 | 78.1±2.9 | 0.729 |
| Male sex | 3 (33%) | 18 (41%) | 0.488 |
| Body weight, kg | 58.0±9.9 | 56.0±10.1 | 0.586 |
| Body mass index, kg/m^2^ | 23.8±3.3 | 22.9±3.4 | 0.508 |
| Duration of AF, years | 1.0 (0.3–6.5) | 0.8 (0.3–3.5) | 0.794 |
| AF type |  |  |  |
| Paroxysmal | 6 (67%) | 31 (71%) | 0.554 |
| Persistent | 3 (33%) | 13 (30%) | 0.554 |
| Long-standing persistent | 1 (11%) | 1 (2.3%) | 0.313 |
| Antiarrhythmic drugs |  |  |  |
| Class I | 1 (11%) | 13 (30%) | 0.242 |
| Class III | 4 (44%) | 7 (16%) | 0.076 |
| Comorbidity |  |  |  |
| Hypertension | 9 (100%) | 35 (80%) | 0.160 |
| Diabetes mellitus | 6 (67%) | 6 (14%) | 0.002 |
| Coronary artery disease | 3 (33%) | 1 (2.3%) | 0.013 |
| Stroke/TIA | 0 (0%) | 8 (18%) | 0.200 |
| Hemodialysis | 1 (11%) | 1 (2.3%) | 0.313 |
| Echocardiographic data |  |  |  |
| LAD, mm | 41.5±6.3 | 41.5±6.4 | 0.986 |
| LVEDD, mm | 45.8±7.8 | 45.9±4.4 | 0.971 |
| LVEDS, mm | 31.8±8.8 | 31.3±6.7 | 0.833 |
| LVEF, % | 56.7±7.9 | 60.7±9.0 | 0.225 |
| CHADS_2_ score | 3.7±0.5 | 3.2±1.1 | 0.204 |
| CHA_2_DS_2_-VASc score | 5.4±0.7 | 4.8±1.1 | 0.095 |
| Laboratory data |  |  |  |
| Creatinine clearance, mL/min | 40.7±23.5 | 48.9±15.2 | 0.341 |
| BNP levels, pg/dL | 252 (123–814) | 167 (123–294) | 0.169 |
| DOAC | 7 (78%) | 37 (84%) | 0.480 |
| History of device implantation |  |  |  |
| Pacemaker | 1 (11%) | 7 (16%) | 0.589 |
| ICD | 2 (22%) | 0 (0%) | 0.026 |
| CRT | 0 (0%) | 0 (0%) | NA |
| Medications |  |  |  |
| ACEI/ARB | 5 (56%) | 22 (50%) | 0.525 |
| ARNI | 0 (0%) | 0 (0%) | NA |
| Beta-blocker | 7 (78%) | 25 (57%) | 0.216 |
| Spironolactone | 3 (33%) | 6 (14%) | 0.169 |
| SGLT-2 inhibitor | 0 (0%) | 0 (0%) | NA |
| Diuretic | 5 (56%) | 16 (36%) | 0.240 |
| Etiology of HF |  |  |  |
| Tachycardia-induced cardiomyopathy | 3 (33%) | 33 (75%) | 0.023 |
| Ischemic cardiomyopathy | 3 (33%) | 4 (9.1%) | 0.086 |
| Dilated cardiomyopathy | 0 (0%) | 1 (2.3%) | 0.830 |
| Hypertrophic cardiomyopathy | 0 (0%) | 1 (2.3%) | 0.830 |
| Valvular heart disease | 1 (11%) | 2 (4.5%) | 0.435 |
| Sarcoidosis | 0 (0%) | 0 (0%) | NA |
| Amyloidosis | 1 (11%) | 0 (0%) | 0.170 |
| Other | 1 (11%) | 3 (6.8%) | 0.536 |
| Session time, min (from puncture to session end) | 148±28.5 | 131±30.0 | 0.154 |
| Major complications | 1 (11%) | 4 (9.1%) | 0.622 |

Abbreviations are as in Supplemental Table 4.

**Supplemental Table 13.** Comparison of post-ablation HF medication use between older and younger patients

|  | Patients with HF and AF  (n=549) | Patients with HF aged ≥75 years  (n=185) | Patients with HF aged <75 years  (n=364) | *p* value^*^ |
| --- | --- | --- | --- | --- |
| Medications |  |  |  |  |
| ACEI/ARB | 256 (47%) | 82 (44%) | 174 (48%) | 0.456 |
| ARNI | 8 (1.5%) | 2 (1.1%) | 6 (1.6%) | 0.458 |
| Beta-blocker | 334 (61%) | 105 (57%) | 229 (63%) | 0.172 |
| Spironolactone | 108 (20%) | 42 (23%) | 66 (18%) | 0.197 |
| SGLT-2 inhibitor | 28 (5.1%) | 7 (3.8%) | 21 (5.8%) | 0.318 |
| Diuretic | 175 (32%) | 77 (42%) | 98 (27%) | <0.001 |

ACEI, angiotensin-converting enzyme inhibitor; AF, atrial fibrillation; ARB, angiotensin receptor blocker; ARNI, angiotensin receptor neprilysin inhibitor; HF, heart failure; SGLT, sodium glucose co-transporter. ^*^ ≥75 years vs. <75 years.

**Supplemental Figure 1**


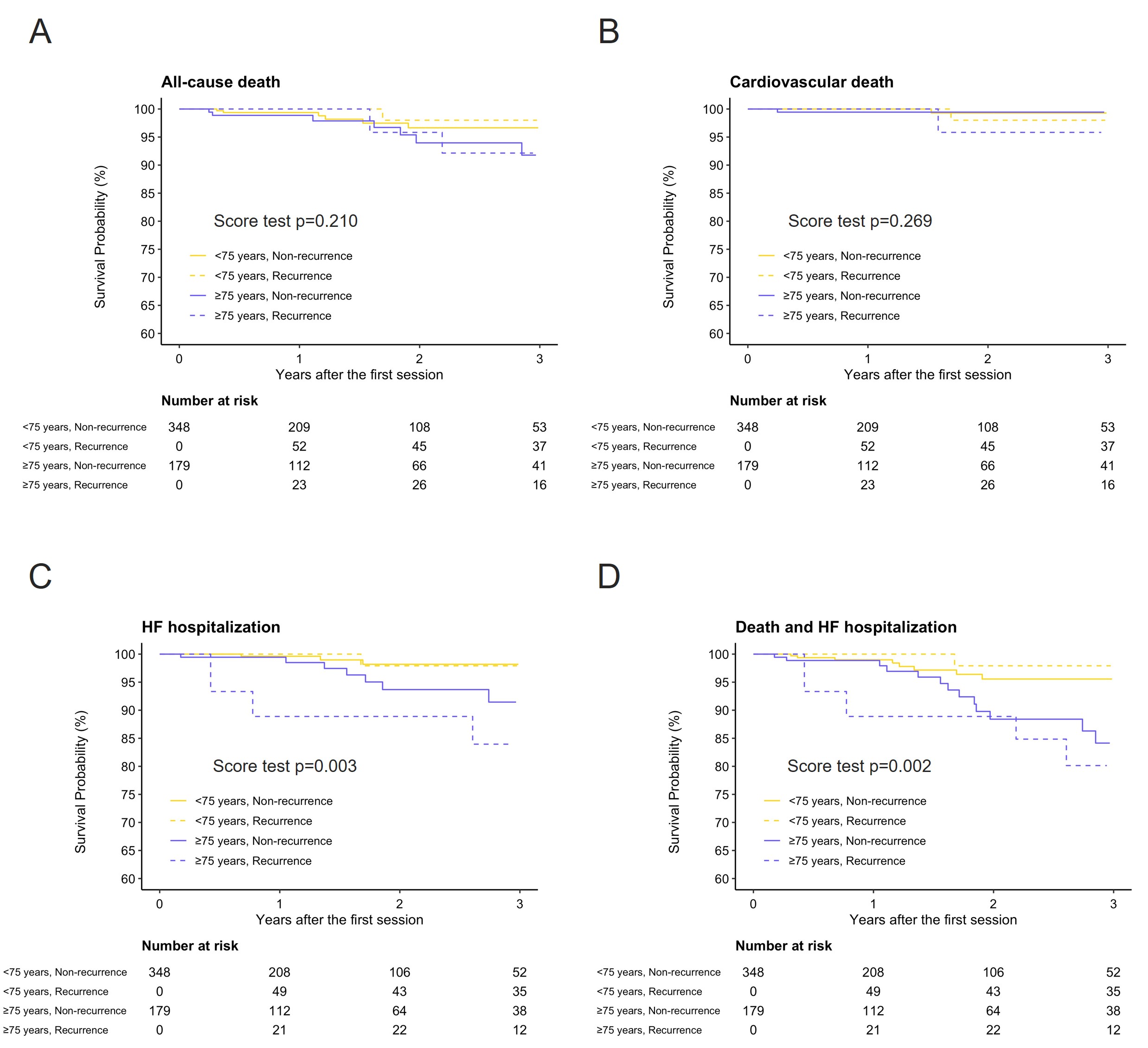


***Supplemental Figure 1****.* Survival curves using the Simon-Makuch analysis after adjusting for a time-dependent event, recurrence factor between patients with HF aged ≥75 or <75 years.

All-cause death (A), cardiovascular death (B), HF hospitalization (C), and death and HF hospitalization (D).

AF, atrial fibrillation; HF, heart failure.

**Supplemental Figure 2**


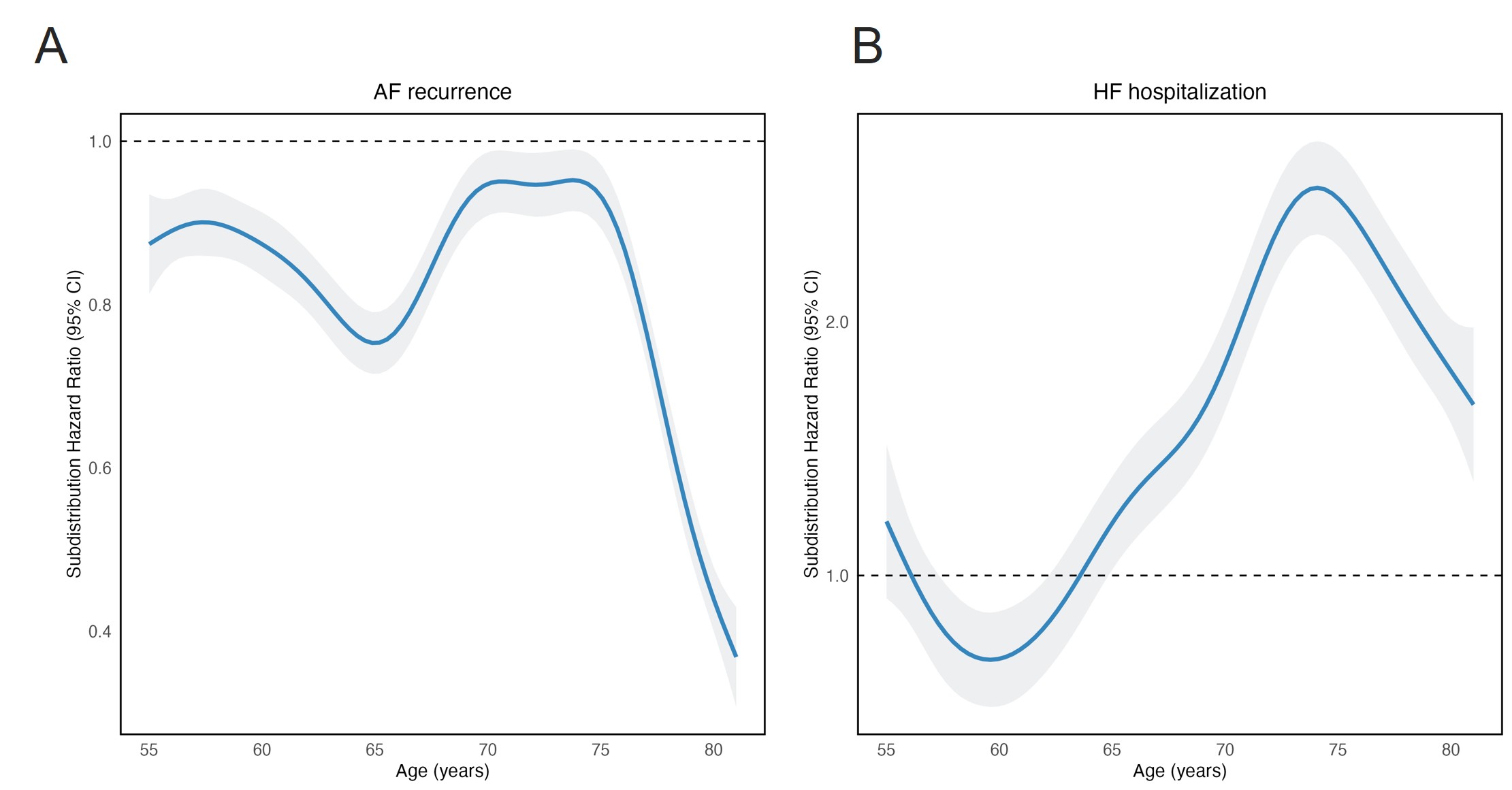


***Supplemental Figure 2****.* Estimated risk of outcomes after cryoablation according to age, accounting for the competing risk of death

The estimated risks of adverse events were analyzed with age as a continuous variable. The solid blue line illustrates the estimated hazard ratio, with 95% CIs depicted as shaded areas.

AF, atrial fibrillation; CI, confidence interval; HF, heart failure.

**Supplemental Figure 3**


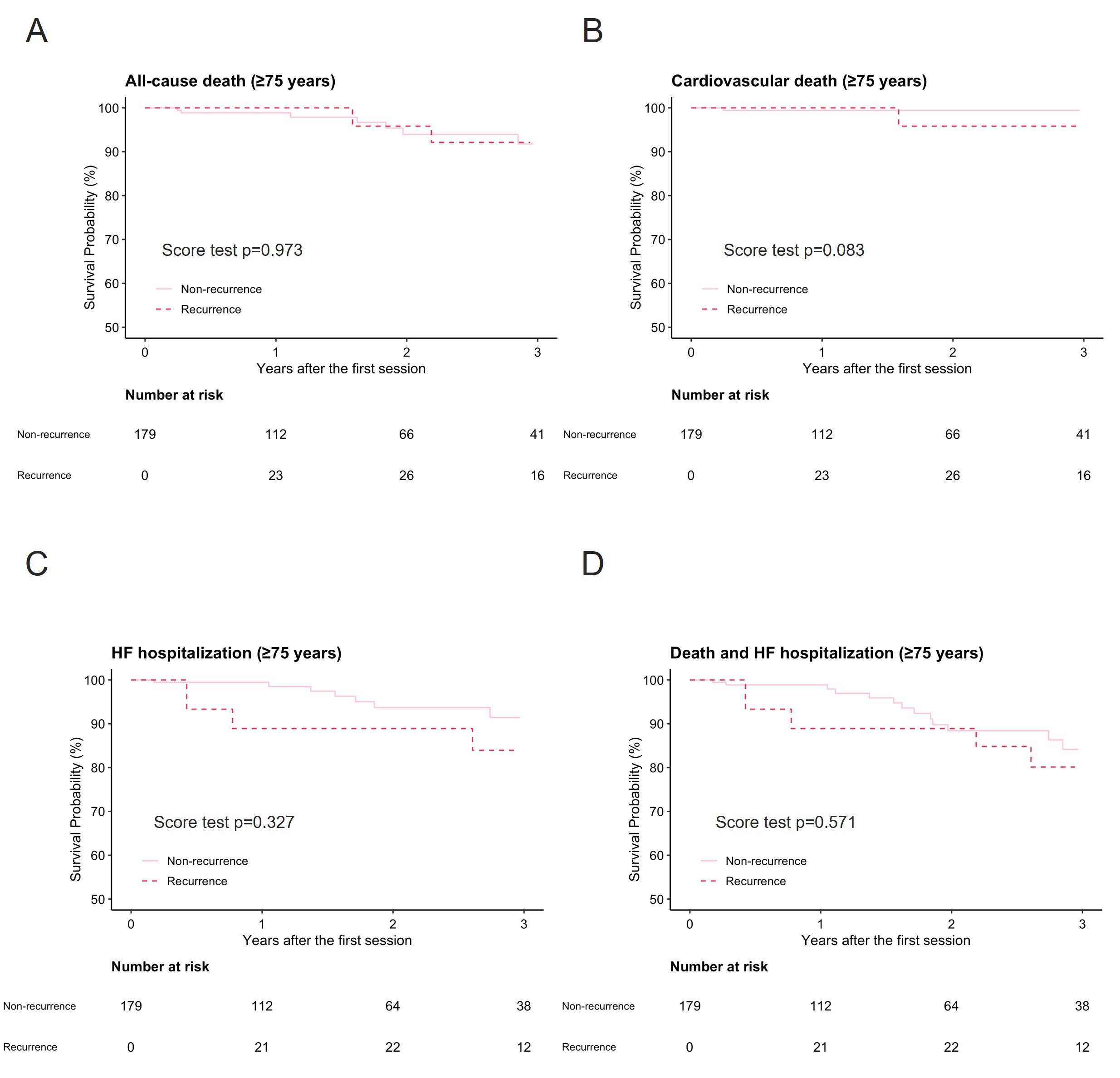


***Supplemental Figure 3****.* Survival curves using Simon-Makuch analysis after adjusting for a time-dependent event; recurrence factor in older patients with or without recurrence.

All-cause death (A), cardiovascular death (B), HF hospitalization (C), and composite endpoint (D). HF, heart failure.

**Supplemental Figure 4**

**
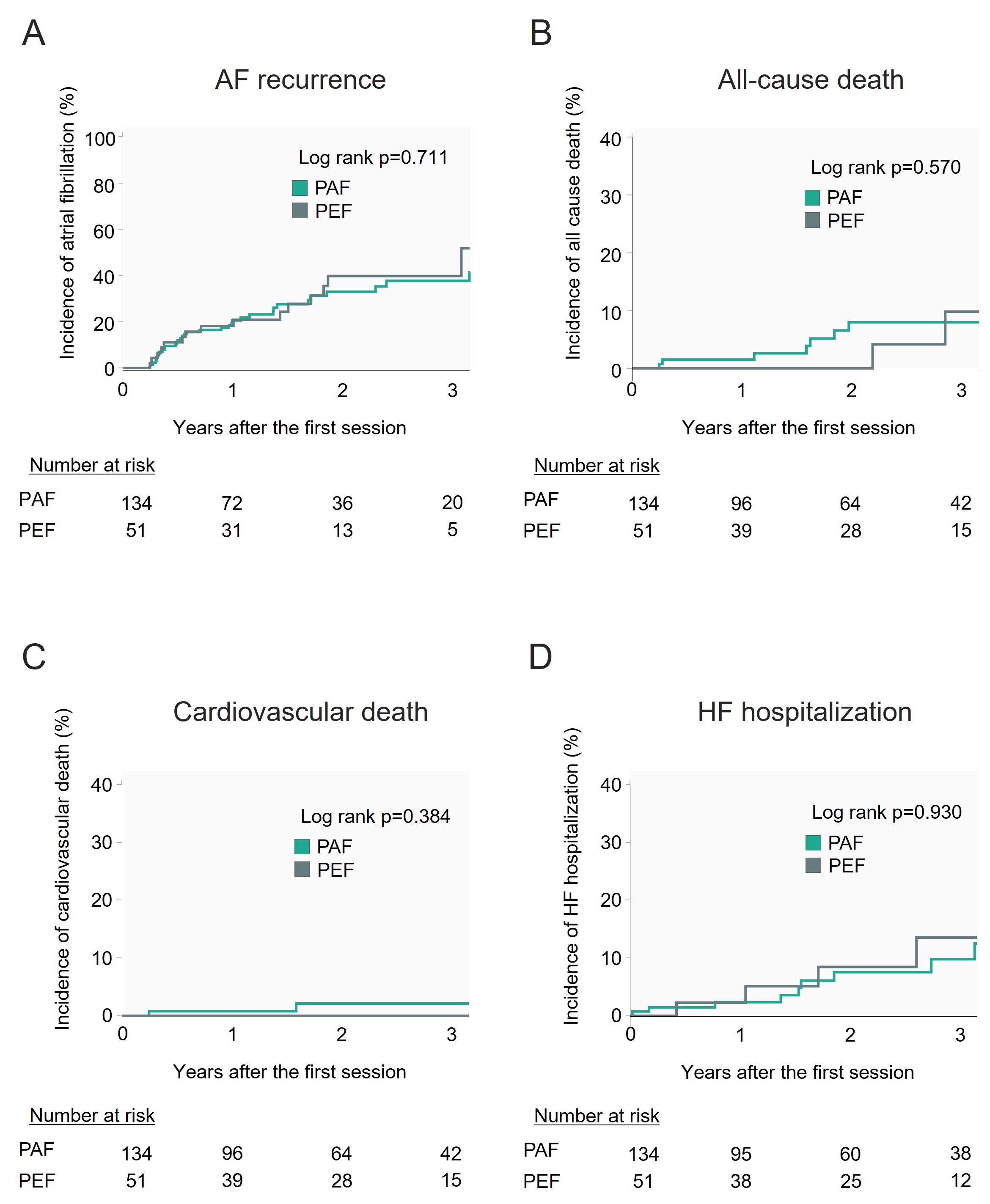
**

***Supplemental Figure 4****.* Kaplan–Meier incidence curves of AF recurrence (A), all-cause death (B), cardiovascular death (C), and HF hospitalization (D) between patients with PAF and PEF. AF, atrial fibrillation; HF, heart failure; PAF, paroxysmal atrial fibrillation; PEF, persistent atrial fibrillation.

**Supplemental Figure 5**


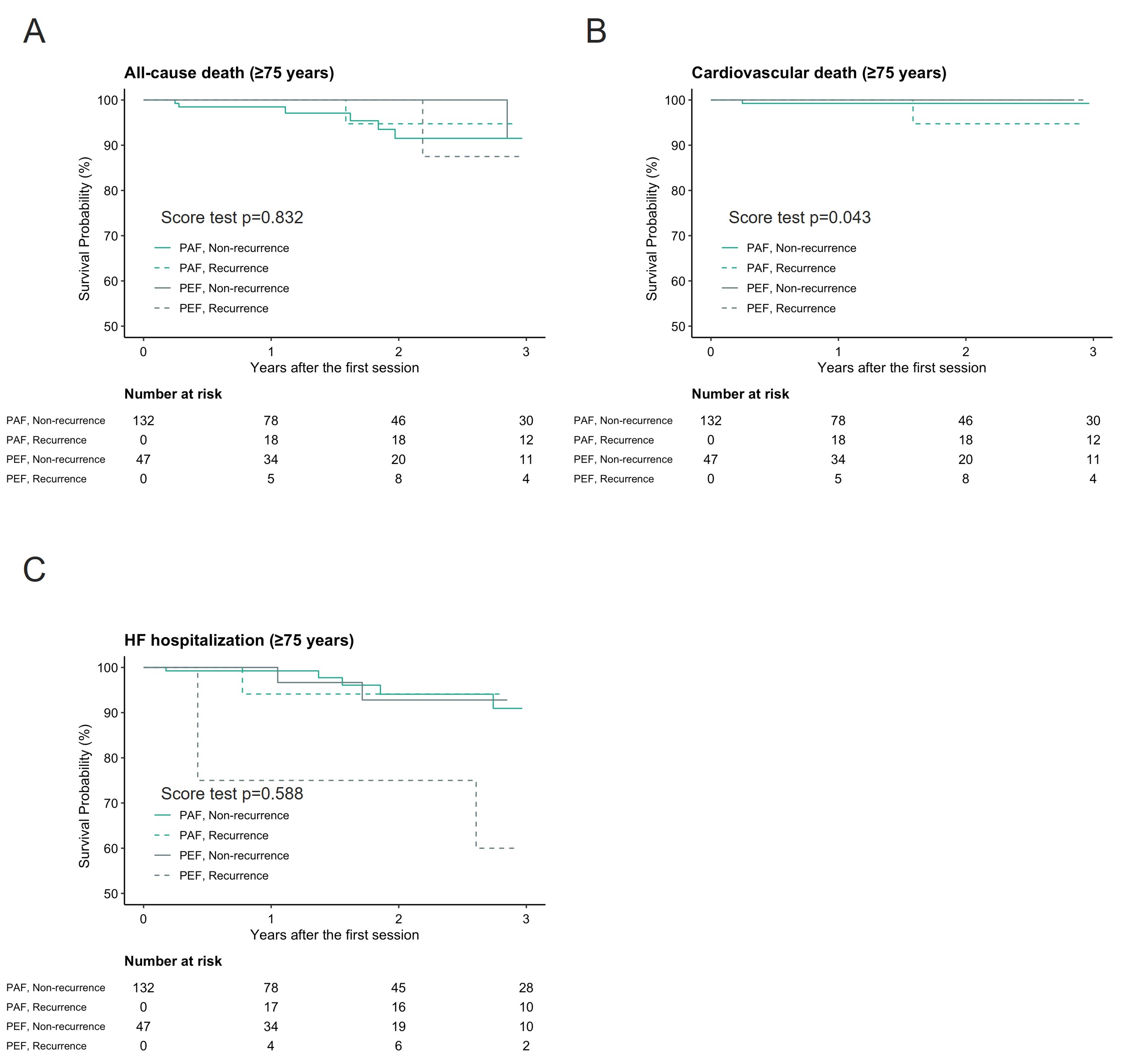


***Supplemental Figure 5****.* Survival curves using Simon-Makuch analysis after the adjustment of a time-dependent event, recurrence factor between older patients with PAF and PEF.

AF, atrial fibrillation; HF, heart failure; PAF, paroxysmal atrial fibrillation; PEF, persistent atrial fibrillation.

**Reference**

1. Akaike H. A new look at the statistical model identification. *IEEE Transactions on Automatic Control* 1974;19:716-23.
